# Using Structure-guided Fragment-Based Drug Discovery to Target *Pseudomonas aeruginosa* Infections in Cystic Fibrosis

**DOI:** 10.3389/fmolb.2022.857000

**Published:** 2022-03-30

**Authors:** Sheikh Mohammed Arif, R. Andres Floto, Tom L. Blundell

**Affiliations:** ^1^ Department of Biochemistry, University of Cambridge, Cambridge, United Kingdom; ^2^ Molecular Immunity Unit, Department of Medicine University of Cambridge, MRC-Laboratory of Molecular Biology, Cambridge, United Kingdom; ^3^ Cambridge Centre for Lung Infection, Royal Papworth Hospital, Cambridge, United Kingdom

**Keywords:** FBDD, fragment-based drug discovery, antibiotics, cystic fibrosis, *Pseudomonas*, anti-virulence

## Abstract

Cystic fibrosis (CF) is progressive genetic disease that predisposes lungs and other organs to multiple long-lasting microbial infections. *Pseudomonas aeruginosa* is the most prevalent and deadly pathogen among these microbes. Lung function of CF patients worsens following chronic infections with *P. aeruginosa* and is associated with increased mortality and morbidity. Emergence of multidrug-resistant, extensively drug-resistant and pandrug-resistant strains of *P. aeruginosa* due to intrinsic and adaptive antibiotic resistance mechanisms has failed the current anti-pseudomonal antibiotics. Hence new antibacterials are urgently needed to treat *P. aeruginosa* infections. Structure-guided fragment-based drug discovery (FBDD) is a powerful approach in the field of drug development that has succeeded in delivering six FDA approved drugs over the past 20 years targeting a variety of biological molecules. However, FBDD has not been widely used in the development of anti-pseudomonal molecules. In this review, we first give a brief overview of our structure-guided FBDD pipeline and then give a detailed account of FBDD campaigns to combat *P. aeruginosa* infections by developing small molecules having either bactericidal or anti-virulence properties. We conclude with a brief overview of the FBDD efforts in our lab at the University of Cambridge towards targeting *P. aeruginosa* infections.

## Introduction

Cystic fibrosis (CF) is a life-shortening autosomal-recessive Mendelian disease affecting approximately 100,000 people worldwide (Cystic Fibrosis Foundation, 2017 Annual Data Report, 2021; United Kingdom; Cystic Fibrosis Registry 2020 Annual Data Report, 2021). In the 1950s, the majority of the CF patients did not live beyond infancy (Davis, 2006; Elborn, 2016). Four decades later, the life expectancy had improved to 31 years (Cystic Fibrosis Foundation. 2017. Cystic Fibrosis Foundation patient registry. 2016 annual data report. Cystic Fibrosis Foundation, Bethesda, MD.). At present, with further improvement in the diagnosis and treatment, the median survival age of CF patients has increased to 50.6 years in United Kingdom (UK Cystic Fibrosis Registry 2020 Annual Data Report, 2021) and 59 years in United states (UK Cystic Fibrosis Registry 2020 Annual Data Report, 2021). The majority of morbidity and mortality in CF is caused by chronic bacterial lung infections (Elborn, 2016; Molina and Hunt, 2017). CF is caused by bi-allelic deleterious mutations in the Cystic Fibrosis Transmembrane Conductance Regulator (CFTR) protein, leading to defects in chloride and bicarbonate ion transport across epithelial surfaces, and consequently the production of thick secretions that disrupt mucociliary clearance in the lungs, predisposing to chronic bacterial infections, and progressive inflammatory lung disease.

The most important bacterial infection in CF is *P. aeruginosa*, a Gram-negative bacterium that, in addition to causing opportunistic and hospital-acquired infections, can cause chronic respiratory infections in individuals with underlying inflammatory lung disease, including CF. Approximately 40% of the adult CF population in the United Kingdom is currently chronically infected with *P. aeruginosa* (2019 CF registry), leading to increased mortality and morbidity (Flume et al., 2007; Crull et al., 2018) associated with the emergence of multi-drug resistant organisms and antibiotic failure.

### Current Treatment Regimens for *P. aeruginosa* Infections and Need for Novel Therapeutics

At present, nine categories of anti-pseudomonal antibiotics are used to treat *P. aeruginosa* infections including penicillin-β-lactamase combinations (piperacillin-tazobactam and ticarcillin-clavulanate), cephalosporins (Ceftazidime, Cefepime, Cefoperazone and Cefiderocol), a monobactam (aztreonam), fluoroquinolones (Ciprofloxacin, Levofloxacin, Prulifloxacin, Delafloxacin and Finafloxacin), a phosphonic acid derivative (Fosfomycin), carbapenems (Doripenem, Imipenem/cilastatin and Meropenem), novel β-lactams with β-lactamase inhibitors (ceftazidime–avibactam, ceftolozane/tazobactam, Imipenem/cilastatin–relebactam, Meropenem–vaborbactam), aminoglycosides (Tobramycin, Gentamicin, Amikacin and Plazomicin) and polymyxins (Colistin and PolymyxinB) (Ibrahim et al., 2020).

*P. aeruginosa* is equipped with a high level of intrinsic antibiotic resistance owing to restricted outer membrane permeability, efflux systems that pump antibiotics out of the cell and production of antibiotic-inactivating enzymes such as β-lactamases. *P. aeruginosa* can also readily acquire antibiotic resistance through mutations and acquisition of resistance plasmids (Potron et al., 2015; Pang et al., 2019), leading to the emergence of multidrug-resistant (MDR), extensively drug-resistant (XDR) and pandrug-resistant (PDR) strains of *P. aeruginosa* (El Zowalaty et al., 2015; Bassetti et al., 2018). In addition, *P. aeruginosa* possesses adaptive resistance mechanisms against antibiotics, including biofilm-mediated resistance and the formation of multidrug-tolerant persister cells which further limit the effectiveness of current antibiotic treatments (Pang et al., 2019). As a consequence this pathogen is listed in the “critical” category of antibiotic-resistant “priority pathogens” published recently by WHO (WHO, 2017) (Shrivastava et al., 2018). Hence, there is an urgent need for new antibiotics along with the discovery and development of novel potential therapeutic strategies such as quorum sensing inhibition, lectin inhibition, iron chelation, phage therapy, vaccine strategy, nanoparticles, antimicrobial peptides and electrochemical scaffolds, which present new avenues against *P. aeruginosa* infections (Pang et al., 2019).

### Fragment-Based Drug Discovery Approach and Work Flow

Structure-guided fragment-based drug discovery (FBDD) is a powerful approach, now widely used both in academia and industries to produce novel high-quality drug-like molecules (Blundell et al., 2002; Murray and Rees, 2009; Scott et al., 2012; Mashalidis et al., 2013; Erlanson et al., 2016). This approach involves screening a library consisting of small “fragments” (MW < 300 Da) of drug-like molecules against a defined target protein, using various biophysical, biochemical and structural biology methods. Compared with the traditional high-throughput screens of drug-sized molecules, the binding and subsequent growth of low molecular weight fragments to drug-like compounds allows a more extensive exploration of chemical space even when using small libraries and can lead to superior molecules. Although fragments are usually weak binders, they can bind to hotspots that allow well-defined interactions with the target protein. These can be entropically favourable due to displacement of previously organised bound water molecules (Radoux et al., 2016). The three-dimensional binding mode of these fragments is explored by determining the structure of the fragment-bound target using X-ray crystallography, NMR spectroscopy or cryoelectron microscopy, and guided by these structures the fragments are chemically “grown” or “linked” to form a larger molecule with higher affinity and drug-like properties. Over the past 20 years, FBDD has achieved significant success by delivering six FDA approved drugs namely, vemurafenib (Bollag et al., 2012), venetoclax (Souers et al., 2013), erdafitinib (Perera et al., 2017), pexidartinib (Tap et al., 2015), sotoracib (Blair, 2021) and Asciminib (Eşkazan, 2021) (Table 1). In addition, more than 40 molecules discovered by FBDD are currently in active clinical development (Erlanson, 2021). Four of these molecules: capivasertib (AstraZeneca/Astex/CRUK), lanabecestat (Astex/AstraZeneca/Lilly), pelabresib (CP-0610) (Constellation) and verubecestat (Merk) have reached in the phase 3 of the clinical trial (Erlanson, 2021). However, the use of FBDD for the development of anti-pseudomonal molecules has been limited to only a handful of studies, some of which will be discussed later in this review, but focusing on the contribution of our own lab.

**TABLE 1:** Drugs on the market developed by FBDD approach and their biological targets.

Drug	Biological target	Description
Vemurafenib 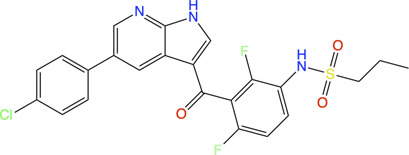	BRAF V600E mutant	Vemurafenib, the first drug developed using FBDD, marketed as Zelboraf, is an inhibitor of B-raf enzyme that lead to programmed cell death in melanoma cell lines
Venetoclax 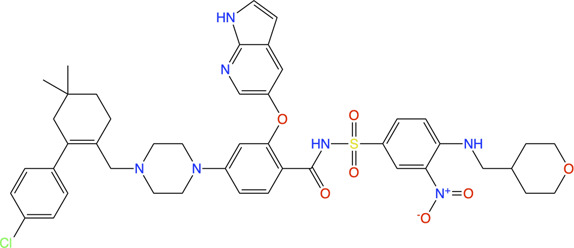	Bcl-2	Venetoclax is a BCL2 homology domain 3 (BH3) mimetic that blocks the anti-apoptotic B-cell lymphoma-2 (Bcl-2) protein leading to programmed cell death of chronic lymphocytic leukemia (CLL) cells
Erdafitinib 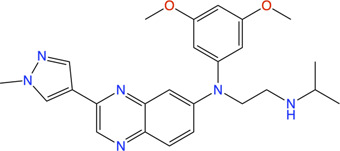	FGFR	Marketed under the brand name Balversa, erdafitinib is a small molecule inhibitor of fibroblast growth factor receptor (FGFR, a subset tyrosine kinase) used for the treatment of bile duct cancer, gastric cancer and esophagial cancer
Pexidartinib 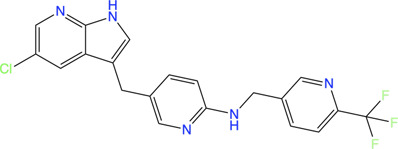	CSF-1R	Marketed as Turalio, pexidartinib is kinase inhibitor that blocks the activity of colony-stimulating factor-1 receptor (CSF-1R). It is used to treat of adults with asymptomatic tenosynovial giant cell tumor (TGST)
Sotorasib 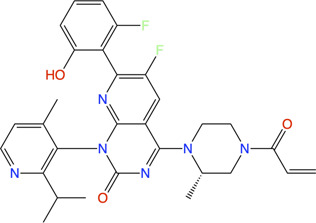	KRAS G12C mutant	Sold under the brand name Lumakras and Lumykras, Sotorasib targets the common mutation G12C in KRAS protein associated with various forms of cancers. It is an inhibitor of RAS gtpase family of protein and used to treat non-small-cell lung cancer (NSCLC)
Asciminib 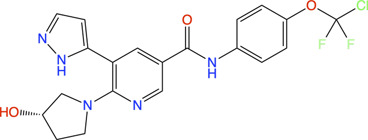	Bcr-ABL	Asciminib (Scemblix) is a protein kinase inhibitor which specifically targets the ABL myristoyl pocket of the fusion protein Bcr-ABL. It is used to treat Philadelphia chromosome-positive chronic myeloid leukemia (Ph + CML)

Until recently one of the main limitations of fragment-based approaches was the need to have crystals with sufficient resolution to see the fragments. This is now less of a problem as many companies such Astex and Astra Zeneca, as well as our own academic work, has been radically transformed by the use of cryo-EM. The power of cryo-EM to visualise small molecules is illustrated by recent work in both our academic work and Astex company work, where cryo-EM is now being widely used for fragment-based drug discovery. A recent example from our academic work is on visualising small drug molecules in the 4,000 amino acid DNA-PKcs published in Nature (Liang et al., 2022).

**FIGURE 1:**
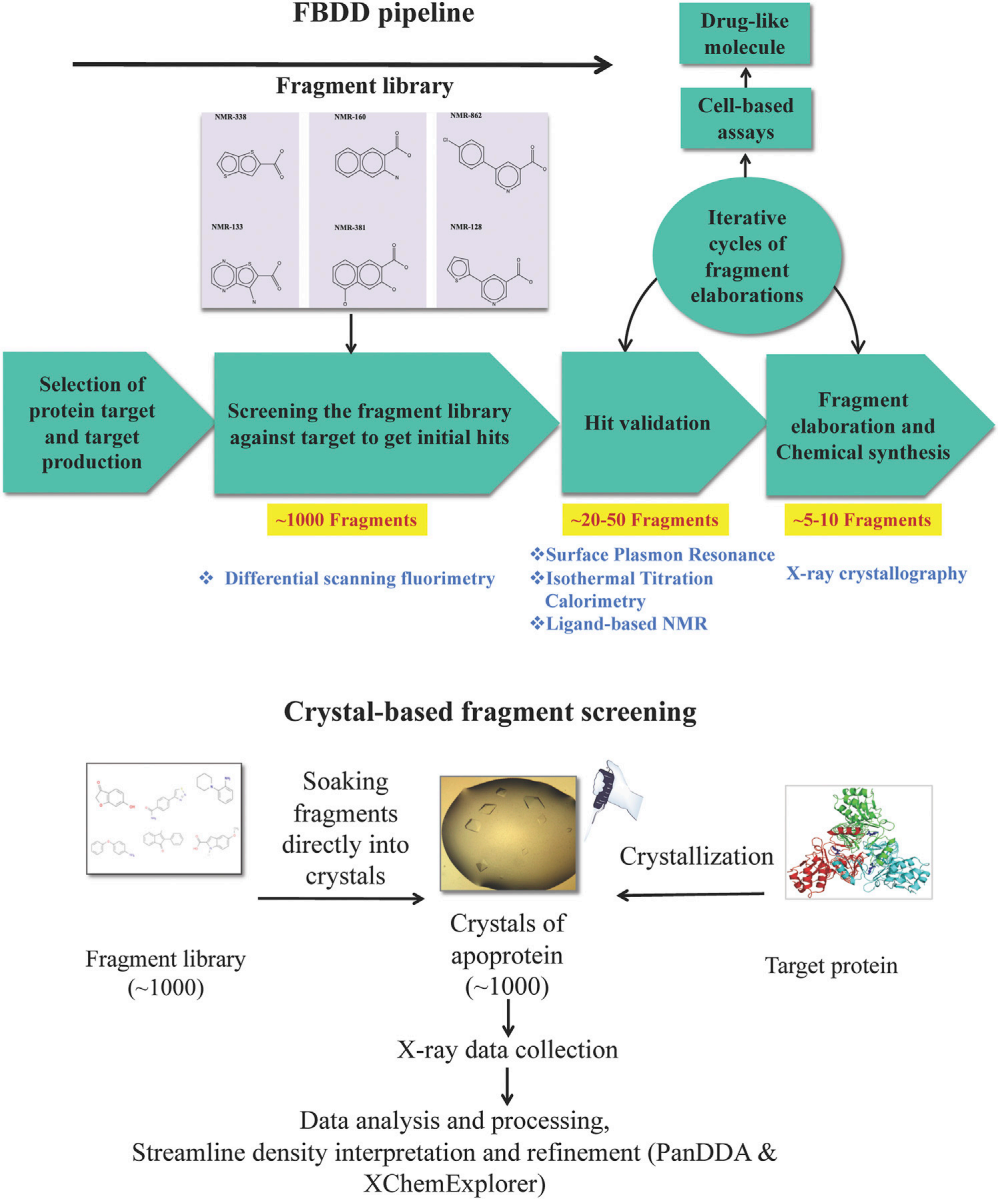
Fragment-based drug discovery (FBDD). The various biochemical, biophysical and structural biology techniques used in a FBDD pipeline are illustrated.

We now review the steps involved in a typical target-oriented structure-guided fragment based drug discovery (Figure 1).

#### Target Selection

Target selection is the first step in our drug discovery pipeline. A systematic analysis of literature on gene essentiality is performed in order to select essential drug-targets. These studies include various molecular genetic approaches, such as gene trapping, homologous recombination, transposon insertion mutagenesis (Van Opijnen and Camilli, 2013; Chao et al., 2016), and more recently developed CRISPR/CAS9 gene editing system (Wang et al., 2015; Evers et al., 2016; Morgens et al., 2016; Senturk et al., 2017; Bartha et al., 2018; You et al., 2020), to identify essential genes by first incorporating inactivating mutations in them and then determining the viability of the cells or organism carrying these mutations. The next step is to look for the similarity of these essential genes to those of the human genome. Targets similar to a gene in the human genome or human gut microbiome are not pursued in order to avoid cross-reactivity of the developed antibiotic and maintain the health of the microbiome. The protein products of these genes are considered for selecting a target. This is followed by the ligandability/druggability analysis by detecting and evaluating the ligand binding sites using modelling programs, such as the α-shape based approach (Edelsbrunner and Mücke, 1994) in MOE (MOLECULAR OPERATING ENVIRONMENT, 2016 Integrated Computer-Aided Molecular Design Platform), SiteMap (Halgren, 2009), LIGSITE (Huang and Schroeder, 2006), CASTp (Dundas et al., 2006) and more recently by generating fragment HOTSPOT maps (Radoux et al., 2016), taking advantage of the available 3-dimensional structure for potential target proteins. Cryptic binding sites can be detected by molecular dynamics simulations (Kuzmanic et al., 2020) and the cosolvent mapping method (Yang and Wang, 2010). In cases where the 3-dimensional structures are not available, they can be modelled using software such as Modeller (Šali and Blundell, 1993; Eswar et al., 2006) and more recently developed deep-learning algorithms, such as AlphaFold (Senior et al., 2020), AlphaFold2 (Jumper et al., 2021) and RoseTTAFold (Baek et al., 2021), and the resulting structure can be then analysed for ligandability. Other criteria while selecting a target include preference for non-membrane proteins and availability of well-established biochemical/functional assays in order to achieve a speedy drug-discovery process.

#### Target Protein Production

Once the target protein is selected, the corresponding gene is cloned into an expression vector using molecular biology techniques such as PCR (Mullis, 1990; Garibyan and Avashia, 2013; Hoseini and Sauer, 2015), restriction digestion (Roberts, 2005) and ligation (Cohen et al., 1973) resulting in an expression plasmid. The target protein is often overexpressed in *Escherichia coli* using this expression plasmid. The overexpressed protein is purified in large quantity using protein chemistry techniques such as affinity chromatography (Rodriguez et al., 2020), ion-exchange chromatography (Walls and Walker, 2017) and size exclusion chromatography (Burgess, 2018; Held and Kilz, 2021).

#### Design/Selection of Fragment Library for a Selected Target

Designing/selecting a generic fragment library for a fragment-screening campaign in general involves considerations such as diversity, solubility, molecular weight (MW), cLogP, polar surface area (PSA), Fsp3 (Lovering et al., 2009; Wei et al., 2020), natural product-likeness and the number of the fragments (Mahmood and Ramachandraiah, 2021). On the other hand, there are certain criteria that are important to consider while assembling/choosing a fragment library with high chemical and structural diversity to be used for screening against a selected target. These involve ligand and protein structure-based approaches of library design/selection (Schuffenhauer et al., 2005). The ligand-based approach involves the knowledge of binding mode of known ligands for the target and the fragments that can undergo similar interactions with the target are selected. Importantly, such interactions are often conserved over the whole target family and hence a fragment identified using this approach can be used to target other members of the family. An example of such approach include selection of metal chelators in a fragment screening campaign against gelatinase (Wang et al., 2002). Commercial fragment libraries dedicated for matrix metalloprotease (MMPs) such as Chelator Fragment Libraries (CFL 1.1), comprising a range of metal chelating moieties (Agrawal et al., 2008) and protease inhibitor-enriched library (Baell and Holloway, 2010), have been deliberately chosen to target a matrix metalloproteinase, LasB (Garner et al., 2012; Kany et al., 2018a). Another example of target-focussed libraries are halogen-enriched fragment libraries (HEFLibs), comprising chemical probes that identify halogen bonds as the main feature of binding mode, and originally constructed to find chemical moieties that stabilise p53 mutants (Heidrich et al., 2019). Structure-based approaches for design/selection of the fragment library may involve in silico screening as described for DNA gyrase ligands (Boehm et al., 2000). The hit rates in fragment screening with such target-focused fragment libraries are much higher than those with generic fragment libraries.

#### Fragment Screening to Identify Hits

Biophysical, biochemical, structural biology and computational methods are used to screen a library of fragments (∼1,000) against the target. The most popular biophysical method used for screening is differential scanning fluorimetry (DSF), often known as thermal shift assay, a technique that measures the denaturation temperature (melting temperature, Tm) of the protein (Senisterra et al., 2012) and allows the detection of compounds that increase the Tm of a target protein on binding by promoting protein stability (Niesen et al., 2007). Surface plasmon resonance (SPR), a label-free technique, is another biophysical technique that is used for direct screening of fragment libraries (Navratilova and Hopkins, 2010). Fragment-screening by SPR is advantageous owing to its cost-effectiveness, the possibility of high-throughput mode and the requirement of a very small amount of protein (Neumann et al., 2007; Chavanieu and Pugnière, 2016). NMR spectroscopy can also be used to perform initial screening, possibly using cocktails of fragments to accelerate the procedure (Leach, 2006).

Microscale thermophoresis (MST), another well-established biophysical technique to quantify any kind of biomolecular interaction (Jerabek-Willemsen et al., 2014; Mueller et al., 2017), is based on thermophoretic mobility, the directed motions of biomolecules and macromolecular complexes in solution in a temperature gradient; these strongly depend on molecular properties such as size, charge, hydration shell or conformation. MST enables the identification of compounds whose binding to a target changes the thermophoretic mobility of the target (Asmari et al., 2018), including even the weak binders, such as fragments (Linke et al., 2016). It has been demonstrated that MST is amenable for implementation into high-throughput screening cascades (Rainard et al., 2018) and has potential to maximize the efficacy of fragment screening campaigns owing to a high degree of automation in the technique generating quantitative data for affinity ranking in a rapid and precise manner (Rainard et al., 2018). Biolayer interferometry (BLI), especially useful for targeting protein–protein interactions (Wartchow et al., 2011), and nanoelectrospray ionization mass spectrometry (ES-MS) (Maple et al., 2012) are other methods that have recently been used for fragment screening and ranking.

Crystal-based screening of a fragment library has also been developed (Patel et al., 2014), and this is routinely practiced at the XChem facility of Diamond Light Source, United Kingdom as a highly streamlined process, allowing more than 1,000 fragments to be screened in less than a week (Douangamath et al., 2020. https://www.diamond.ac.uk/Instruments/Mx/Fragment-Screening.html). For this, many crystals of the target protein are produced and the fragments are soaked in these crystals using the acoustic droplet ejection technique (Collins et al., 2017). High-throughput X-ray diffraction data collection (36 h of unattended beamtime) is carried out on the resulting crystals at the dedicated beamline, IO4-1, of the Diamond Light Source. The processing of X-ray data is performed automatically while the data are being collected, using the dedicated data processing softwares, such as xia2 (Winter 2010) among others. The structure solution and map analysis is performed by the XChemExplorer package (Krojer et al., 2017) including modules such as DIMPLE (Difference Map PipeLinE) for initial refinement and automated difference map calculation to allow for quick assessment of X-ray data to see if a ligand has bound to the structure; AceDRG (Long et al., 2017) for generation of ligand restraints; REFMAC (Murshudov et al., 2011) for refinement; PanDDA (Pearce et al., 2017) for the streamline density interpretation and hit detection; a number of tools from PHENIX (Adams et al., 2010) for structure validation; and Coot (Emsley et al., 2010) for automated model building. The advantage of crystal-based screening is that it can detect fragments that have very weak binding affinities to the target protein.

To cut the cost of experiments and as a pre-screen, virtual docking of the fragment library on the three-dimensional structure of target protein, known as virtual screening (VS), is often performed in order to obtain initial hits (Bielska et al., 2011; Singh et al., 2018; Yamaotsu and Hirono, 2018; Gimeno et al., 2019). However, it is of note that these hits are hypothetical and warrant experimental validation (Zhu et al., 2013). VS is advantageous in cases where a very large library comprising millions of scaffolds has to be screened, where it can reduce the number of molecules needed to be screened experimentally by other biophysical/biochemical methods. VS campaign can be supplemented by machine and deep learning principles, especially when dealing with large data sets (Melville et al., 2009). Machine learning approaches in ligand-based VS can address complex compound classification problems and help predict new active molecules (Lavecchia, 2015). Deep learning in the field has emerged in recent years and it has been demonstrated to have utility beyond bioactivity prediction to other drug discovery problems such as *de novo* molecular design, synthesis prediction and biological image analysis (Chen et al., 2018).

#### Hit Validation

Hits from initial screening are confirmed by ligand-based NMR spectroscopy (Hajduk et al., 1997; Mayer and Meyer, 1999; Dalvit et al., 2001). Real-time fragment-binding affinity and kinetics can be determined using SPR (Yadav et al., 2012; Capelli et al., 2020) and thermodynamics of fragment binding is determined using isothermal titration calorimetry (ITC) (Srivastava and Yadav, 2019). The 3D structures of protein-fragment complexes are determined using X-ray crystallography to instruct the process of fragment elaboration. Functional biochemical assays are performed to measure the inhibition activity of the fragments.

#### Fragment to Lead Optimization and Cell-Based Assays

Guided by the 3D structure of protein-fragment complexes the fragments are elaborated using growing (Hoffer et al., 2018), merging (Nikiforov et al., 2016; Miyake et al., 2019) or linking (Bancet et al., 2020), whichever suits in order to generate compounds with higher affinity and drug-like properties. In-silico molecular modelling is a remarkably beneficial tool as, together with X-ray and cryo-electron microscopy, it provides a means to develop a suitable linker to attach to the low-affinity molecules (Scoffin and Slater, 2015; de Souza Neto et al., 2020). We do this with the help of our chemistry collaborators. The elaborated compounds are then tested by ITC for their affinities to target and functional assays for their inhibition activity against the target. An iterative cycle of fragment growing/merging/linking, followed by biophysical, biochemical and structural analyses while maintaining high ligand efficiency is established. The final compounds, often achieving nanomolar affinities and having potent *in vitro* inhibition, are then used for cell-based assays to check their minimal inhibitory concentrations (MICs) (Kowalska-Krochmal and Dudek-Wicher, 2021). We do this with the help of our cell biologist collaborators.

#### Expected Outcomes

Small “fragments” of drug-like molecules may bind to hotspots on a defined protein target. The initial screening of a library of fragments (∼1,000) often results in several fragments (∼20–50) binding to a target. Hit validation should lead to 5–10 fragments having significant affinity to the target. Fragments can be chemically “grown” or “linked” to deliver compounds with nanomolar binding affinities to the target and altering its activity. Such small-molecule inhibitors developed against essential protein targets can be developed into lead compounds. Further chemical modification of the chemical structure of the lead compound can lead to improvement in potency, selectivity and pharmacokinetic parameters. The pharmacologically active moiety thus obtained may have poor drug likeness which can usually be further modified chemically to result in a more drug-like compound for testing biologically or clinically. Such a compound should be able to compromise the growth and survival of the pathogen inside the host organism.

## Antibiotic Strategies Targeting *P. aeruginosa*

### Targeting Lipopolysaccharide Biosynthesis Pathway

#### Fragment-Based Drug Discovery on LpxA and LpxD

The presence of an outer membrane in Gram-negative bacteria (GNB) protects the bacterial cell not only from the harsh environment but also antibiotics (Koch, 2017). Lipopolysaccharide (LPS), the major component of the outer leaflet of this membrane (Raetz and Whitfield, 2002), are virulence factors essential in many clinically important GNBs, such as *P. aeruginosa,* where it plays important roles in the structural integrity of the bacteria and its defence against the host; hence the enzymes of the LPS biosynthesis pathway are attractive drug targets (Cryz et al., 1984; King et al., 2009). Although there are currently no antibiotics targeting LPS biosynthesis, compounds inhibiting this biochemical pathway can lead to the development of new antibiotics with novel mechanism of action (Jackman et al., 2000; Joo, 2015). Lipid A, a glucosamine disaccharide that is connected to multiple fatty acid chains of various lengths, is the minimal component of LPS required for cellular viability in most GNBs (Anderson et al., 1993; Rotella, 1997; Raetz and Whitfield, 2002). Moreover, lipid A is the antigenic determinant of LPS that triggers septic shock. The enzymes of the LPS biosynthesis pathway, including lipid A biosynthesis, are attractive drug targets for therapeutic interventions. An *in silico* study suggests that, in *P. aeruginosa*, a total of thirteen enzymes are involved in this LPS biosynthesis, of which seven enzymes matched with the list of candidate essential genes obtained by transposon mutagenesis study (Perumal et al., 2007). These enzymes have no human homologues and hence they can serve as potential drug targets.

LpxA, LpxC and LpxD are the first three enzymes in lipid. A biosynthesis pathway. Of these, LpxC has been extensively targeted by antibiotic discovery leading to the development of many small molecule inhibitors with antibacterial properties (Kalinin and Holl, 2017). In contrast, LpxA and LpxD have remained largely unexplored for the development of small molecule inhibitors and are targeted mostly by antibacterial peptides (Williams et al., 2006; Jenkins and Dotson, 2012; Jenkins et al., 2014; Dangkulwanich et al., 2019). LpxA and LpxD are amenable to dual-targeting inhibitors due to their structural similarity and the advantage of such inhibitors include increased potency and reduced likelihood of resistance against the inhibitors (Silver, 2007). FBDD against LpxA and LpxD to develop dual-targeting inhibitor was performed by Kyle G. Kroeck and co-workers (126). An initial fragment screening by virtual docking of a subset of the ZINC fragment library of John J. Irwin and Brian K. Shoichet (Irwin and Shoichet, 2005), followed by SPR characterization of binding fragments and X-ray crystallography analysis of enzyme-fragment complexes to verify binding and to reveal binding modes for lead optimization have identified novel small-molecule scaffolds that can serve as starting point for future inhibitor discovery (Kroeck et al., 2019). Several of these novel ligands, which have shown dual-binding activity (Table 2), have the potential to result in small molecule inhibitors targeting LpxA and LpxD simultaneously. For instance compound 1 in its complex with LpxA and LpxD adopted similar binding poses in the two acyl-chain binding pockets revealing both the structural similarities and differences that can guide the development of future dual-targeting inhibitors. Interestingly, these structures also highlight additional binding hot spots shared by the two enzymes that can be exploited for further lead optimization.

**TABLE 2:** Binding affinities to *Pa*LpxA and *Pa*LpxD of the novel small-molecule scaffolds identified following an FBDD campaign. A few of them show dual binding having affinities to both *Pa*LpxA and *Pa*LpxD. NA: binding affinity could not be determined; NB: No binding.

Compound	Structure	LpxA (μM)	LpxD (μM)
**1**	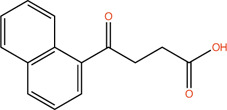	NA	NA
**2**	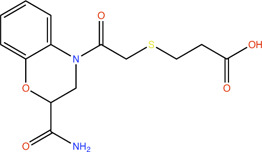	19.5	36.7
**3**	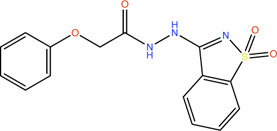	16.7	NB
**4**	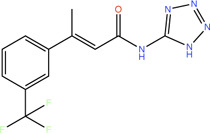	13.6	NB
**5**	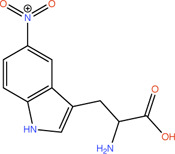	2.1	NB

#### Fragment-Based Drug Discovery on LpxC

The bacterial enzyme UDP-3-O-acyl-N-acetylglucosamine deacetylase (LpxC) is an attractive target for the development of novel therapeutic agents (Erwin, 2016; Chen et al., 2019). It is essential to most Gram-negative bacteria including *P. aeruginosa* (Raetz and Whitfield, 2002, 2002) and catalyzes the removal of an N-acetyl group from UDP-3-O-acyl-N-acetylglucosamine (which constitutes the core of Lipid A), the first committed step in biosynthesis of the LPS, which is an essential component of the bacterial cell wall. Several series of inhibitors have been developed against *P. aeruginosa* LpxC (*Pa*LpxC) employing non-FBDD approach (Erwin, 2016; Kalinin and Holl, 2017; Chen et al., 2019) exploiting the following features of molecules at the binding site: 1) a zinc-chelating motif, 2) a polar group occupying the UDP binding pocket, 3) a linker that is mainly hydrophobic but sometimes makes at least one hydrogen bond interaction, 4) an extended hydrophobic moiety that binds in the tunnel, and 5) often addition of groups out into the solvent at the end of the tunnel to modulate compound properties. The most advanced compounds carry a hydroxamate moiety that coordinates the zinc ion at the core of the enzyme. One of these compounds, ACHN-975 (Cohen et al., 2019; Lpxc et al., 2019), a hydroxamate-based histone deacetylase inhibitor (HDACI) entered clinical trial and was approved by FDA for its use in oncology applications but was discontinued beyond oncology (Lpxc et al., 2019) due to its off-target side effects associated with hydroxamate group (Shen and Kozikowski, 2016).

**FIGURE 2:**
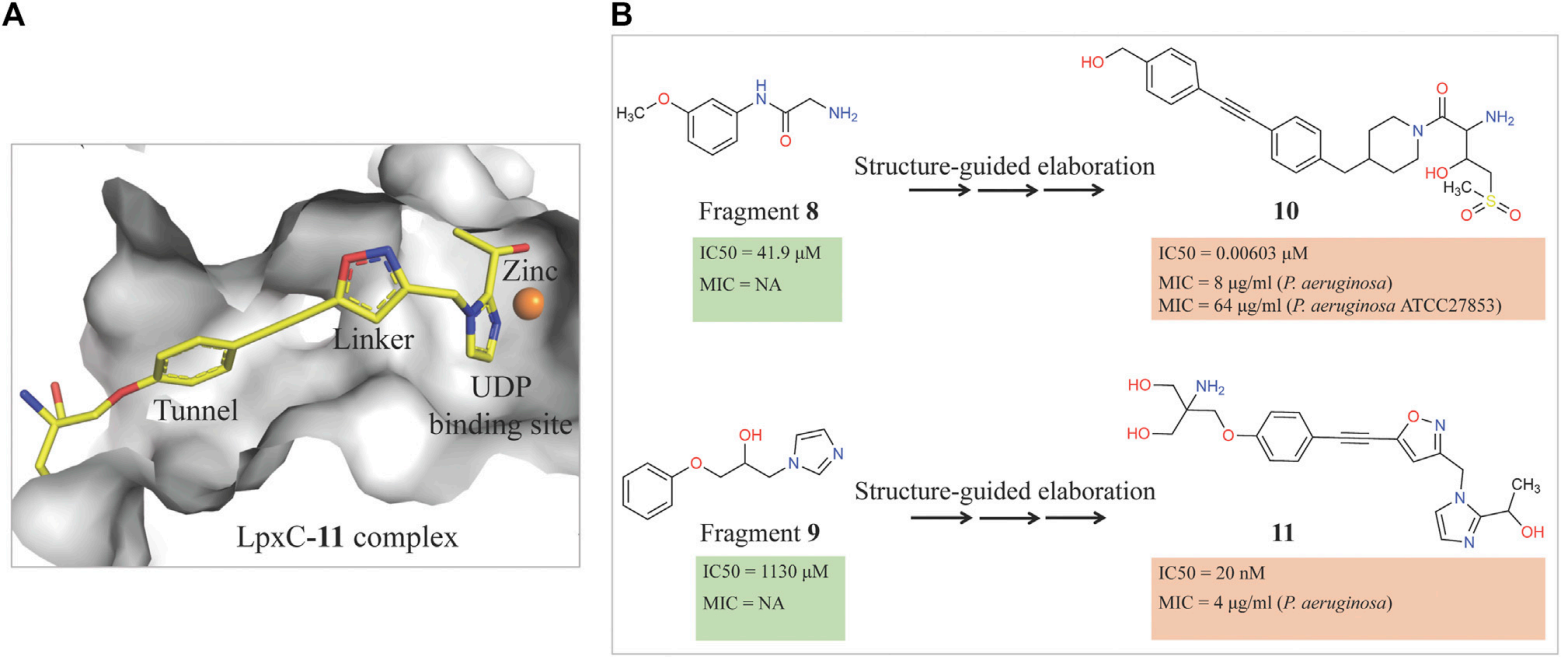
**(A)** Location of compound **11** [2-(1S-hydroxyethyl)-imidazole] in the *Pa*LpxC-**11** complex structure (pdb id 7ci9) determined by X-ray crystallography (Yamada et al., 2020). The protein binding site is represented as grey surface and the compound as stick. The zinc ion is shown as an orange sphere. **(B)** Structure-guided elaboration of fragments **8** and **9**, leading to compounds **10** and **11**, respectively with enhanced potencies and inhibition properties.

Therefore, an FBDD campaign, to identify LpxC inhibitors with a non-hydroxamate metal-coordinating group, was started (Yamada et al., 2020). 1,152 compounds from the Vernalis Research fragment library (Baurin et al., 2004; Chen and Hubbard, 2009) were screened against *Pa*LpxC by ligand-observed NMR (STD, water-LOGSY, and CPMG), using cocktails of 6 fragments (Hubbard et al., 2007; Hubbard and Murray, 2011) followed by singleton NMR competition assays to look for the competitive binding of the fragments with respect to binding of the tool compounds **6** and **7**, (hydroxamate group containing inhibitors reported previously) to LpxC. The 28 fragments showing competitive binding were further assessed in the Fluorescence Polarization (FP) binding assay followed by Fluorescamine-Based Functional Activity Assay. Some of which such as **8** showed clear inhibition with an IC_50_ of 41.9 μM. Two zinc-chelating fragments, a glycine fragment **8** and an imidazole fragment **9**, both of which were competitive with **6**, were selected for further investigation. Both of these were shown to stabilize LpxC in a TSA experiment further confirming the binding. The crystal structure of *Pa*LpxC-fragment **8** complex guided the initial medicinal chemistry and iterative cycle of protein-ligand co-crystallization, confirming binding poses, structure-based modeling predicting the binding poses and guiding the possibility of enhancing potency and chemical modification. This led to the development of a compound **10** with many fold increase in the activity (functional IC_50_ 0.00603 μM) as compared to initial fragment **8** (functional IC_50_ 41.9 μM). However, this compound showed only weak antibacterial activity even in the presence of phenylalanine-arginine β-naphthylamide (PAβN) (an efflux pump inhibitor) (MIC of 8 μg/ml against *P. aeruginosa* and 64 μg/ml against *P. aeruginosa* ATCC27853), so this series was not pursued any further. Similar medicinal chemistry on imidazole fragment **9** resulted in an advanced lead compound 2-(1 Hydroxyethyl) imidazole **11** which exhibited low nanomolar inhibition of *Pa*LpxC (functional IC_50_ of 20 nM) and a minimal inhibitory concentration (MIC) of 4 μg/ml against *P. aeruginosa* (Figure 2). Further optimization and *in vivo* efficacy measurement are under consideration for this compound and will be described in a future publication. The lead compounds of both the series exhibited significant selectivity towards zinc metalloenzyme, LpxC. It was demonstrated that maintaining the zinc-chelation motif as it was in the fragments, engineering aliphatic linkers of appropriate length extending the fragments in the hydrophobic tunnel of LpxC and appending various hydrophobic groups at the para position of the benzyl successfully increased the potencies of compounds with retained ligand efficiencies.

### Role of Aeropath Project in Fragment-Based Drug Discovery

An EU-funded project AEROPATH, coordinated by University of Dundee, was launched in November 2008 with the aim of identifying, characterising and exploiting novel drug targets from the Gram-negative bacterium, *P. aeruginosa*, by applying a multidisciplinary approach encompassing target validation, structural characterization, assay development and hit identification from small molecule libraries following a highthroughput or fragment-based screening campains. Derivation of the structural models of the potential targets was one of the central aims of this project as the structural data allow druggability analysis of the active sites (Krasowski et al., 2011; Radoux et al., 2016) and support the curation of the structure-activity relationship of the identified ligands. Towards the end of this project, 102 targets were selected based on the available genome with preliminary annotation of *P. aeruginosa* strain PAO1 together with gene essentiality studies and other considerations such as feasibility of enzyme assays, chemogenomics information and an appropriate balance of novel uncharacterized proteins versus established targets for antibacterial drug design (Moynie et al., 2013). *De novo* structures of 39 of these targets were determined using X-ray crystallography and NMR. In addition, the structures of more than 60 complexes involving substrate, cofactor and inhibitors have been determined and published by the consortium. Crystal structures of eight targets including both hypothetical uncharacterized protein and metabolic enzymes from various functional classes were reported before the end of the project (Moynie et al., 2013). This plethora of structural information was envisaged to aid the FBDD campaigns.

### Targeting Fatty Acid Biosynthesis Pathway

#### Fragment-Based Drug Discovery to Target FabG

**FIGURE 3:**
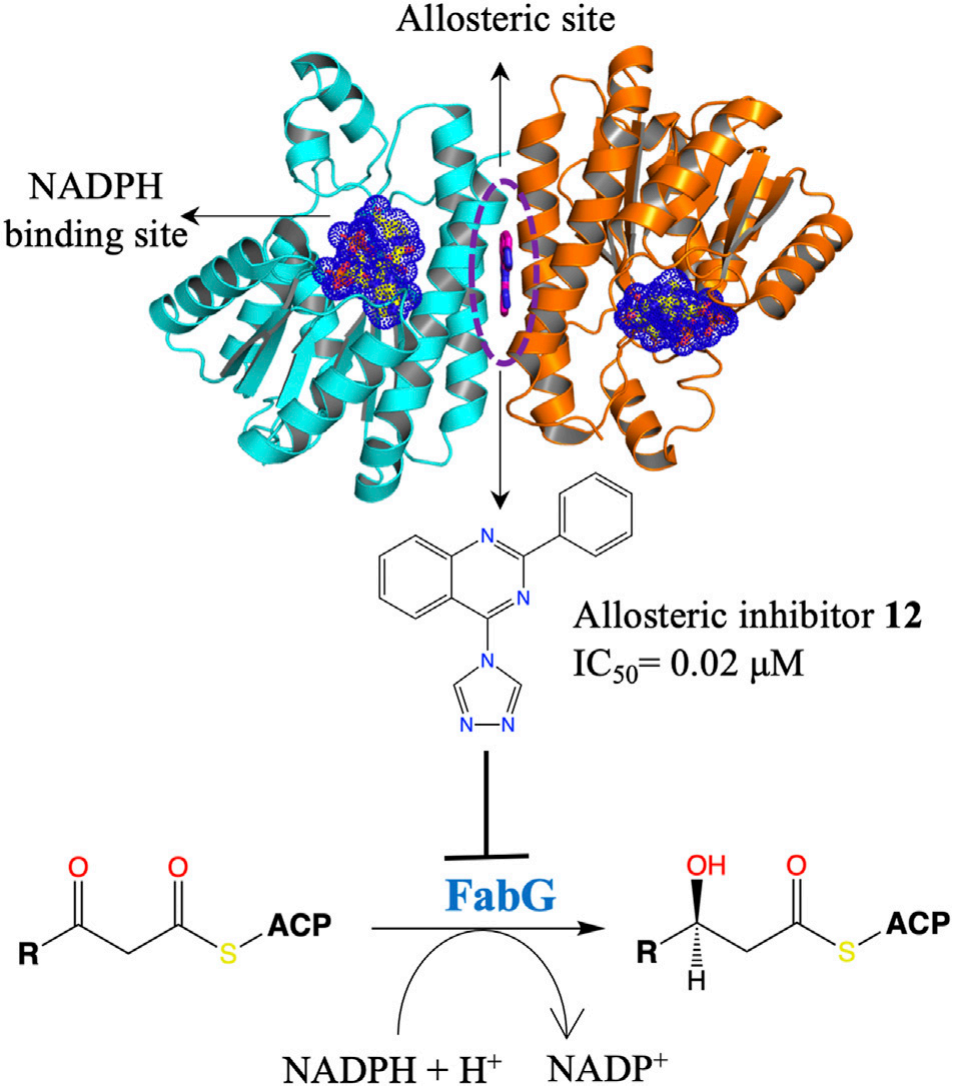
An allosteric inhibitor (**12**) of *Pa*FabG developed by FBDD with an IC_50_ of 0.02 μM. The cosubstrate NADPH from *Pa*FabG-NADPG complex (pdb id 4ag3) is mapped on the *Pa*FabG-FG01 complex structure (pdb Id 4bnu) determined by X-ray crystallography (Cukier et al., 2013). The inhibitor binds at the dimer-dimer interface of *Pa*FabG. Binding of the inhibitor results in disturbance of the binding site of NADPH.

The fatty acid synthesis type II (FAS II) system of bacteria has been identified as an attractive target for therapeutic interventions and several antibiotics targeting this pathway are known, such as triclosan, isoniazid and thiolactomycin (Campbell and Cronan, 2001; Zhang et al., 2004; Heath and Rock, 2006; Parsons and Rock, 2011; Pan et al., 2012). FabG is a NADPH-dependent 3-oxoacyl-acyl carrier protein (ACP) reductase that plays a key role in the FAS II system of pathogenic microorganisms and has been identified as an attractive drug target. It catalyses the reduction of 3-oxoacyl-ACP to 3-D-hydroxyacyl-ACP intermediates during the elongation cycle of fatty acid biosynthesis (Rock and Jackowski, 2002; Goodman and McFadden, 2008; Chan and Vogel, 2010). FabG qualifies to be a promising drug target due to its essentiality, high conservation across bacteria and presence of only a single isoform in most of the bacterial species (Zhang et al., 2004). Inhibitors of *P. aeruginosa* FabG (*Pa*FabG) identified include largely natural product extracts that pose significant drug development challenges and hence none of them have reached the clinic (Zhang and Rock, 2004; Tasdemir et al., 2006; Wickramasinghe et al., 2006; Sohn et al., 2008; Zhang et al., 2008). As part of the AEROPATH project, Cyprian D. Cukier and coworkers have established the essentiality of *fabG* gene in *P. aeruginosa* using gene knockout procedure and following a FBDD approach they then developed a series of novel *Pa*FabG inhibitors with IC_50_ values in nanomolar to low micromolar range and ligand efficiencies in the range of 0.37–0.53 (Cukier et al., 2013). Although the compounds show no phenotypic response in the Gram-negative *P. aeruginosa* either due to poor penetration of the compounds through the Gram-negative cell wall or to rapid efflux of the compounds, the diverse chemotypes of these inhibitors presents a number of options for optimization to increase intracellular concentrations. Structural investigation of 16 *Pa*FabG-inhibitor complexes by X-ray crystallography in this study reveals that inhibitors bind at a novel allosteric site (cryptic binding site) located at the dimer-dimer interfaces of *Pa*FabG (Figure 3) and this binding induces the conformational changes that propagate to the active site and results in the disturbance of the catalytic triads (residues S141, Y154, and K158) with loss in the binding affinity of cosubstrate NADH, thus inhibiting the enzyme. Kinetic analysis of inhibition suggested a noncompetitive mode of inhibition with respect to NADH.

## Anti-Virulence Strategies Against *P. aeruginosa*

Antibiotic development to treat *P. aeruginosa* infections is undergoing a crisis due to the rapid evolution and spread of resistance in bacteria against the current antibiotics. Interfering with bacterial virulence network including virulence factors (proteases, elastase, endotoxins, and polycyanin), lipopolysaccharides, flagella, extracellular polysaccharides, and type II, III, IV and VI secretion system, instead of targeting their viability (growth and survival), to combat *P. aeruginosa* infections offers tantalizing prospects of novel antimicrobials and this approach has gained momentum in recent years to overcome today’s crisis in anitibacterial development (Papaioannou et al., 2013; Anantharajah et al., 2016; Gao et al., 2017; Boulant et al., 2018; Ranjbar et al., 2019). Moreover, this strategy will reduce the propensity to induce resistance as it removes the strong selection pressure imposed by bacteriostatic or bactericidal agents.

### Targetting Quorum-Sensing Systems to Develop “Second Generation” Antibiotics

Inhibiting quorum-sensing systems (QSS) and the regulators that promote biofilm formation is one such novel strategy to attenuate *P. aeruginosa* virulence (O’Loughlin et al., 2013; Shao et al., 2020; Manos, 2021). Four QS pathways (*pqs, iqs, las, rhl*) have been identified in *P. aeruginosa* (Lee et al., 2013). Of these, *pqs* and *las* mediated QSS have been targeted using FBDD and this will be described in the following section of this review.

#### Fragment-Based Drug Discovery on LasB

**FIGURE 4:**
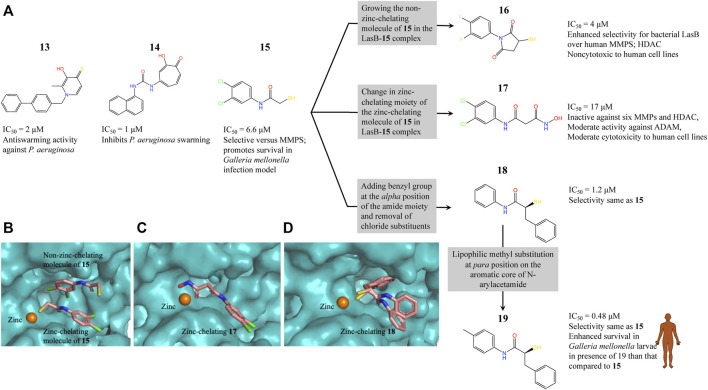
**(A)** Inhibitors developed using FBDD against *Pa*LasB with IC_50_ values in the low micromolar range. Compounds **16**, **17** and **18** were developed using three different strategies guided by the crystallographic complex of *Pa*LasB with **15 (B)**. Compound **19** with best IC_50_ value was obtained from compound **18** by substitution of a hydrophobic group guided by the structure of *Pa*LasB-**18** complex (pdb id 7oc7, unreleased) (Kaya et al., 2021). **(B)** Locations of compound **15** in *Pa*LasB-**15** complex (pdb id 6f8b) determined by X-ray crystallography (Kany et al., 2018a). Two molecules of compound **15** bind in the active site of *Pa*LasB, one of which coordinates with the active site zinc ion. **(C)** *Pa*LasB-**17** complex (pdb id 6fzx) with only one molecule of compound **17** binding and coordinating with zinc ion of *Pa*LasB (Kany et al., 2018b). **(D)** *Pa*LasB-**18** complex (pdb id 7oc7, unreleased) with two conformers of a single molecule of **18**. The inhibitors are shown as sticks and zinc ions as orange spheres in **(A)**, **(B)** and **(C)**. The coordinates for the unreleased pdb 7oc7 was kindly provided by Prof. Dr. Anna K. H. Hirsch.

LasB (or pseudolysin, *Pseudomonas* elastase B) is the most abundant extracellular collagenase protease secreted by *P. aeruginosa* with a hydrolysis activity against a broad spectrum of substrate proteins from the host, causing damage to host tissues, disruption of the host immune response and promoting inflammation associated with *P. aeruginosa* virulence and disease pathology (Wretlind and Pavlovskis, 1983; Saint-Criq et al., 2018; Galdino et al., 2019). Since LasB is extracellular the direct inhibitors are not required to cross the difficult-to-penetrate *P. aeruginosa* cell membrane and the fact that the target belongs to a class of validated drug targets (metalloprotease), against which there are already clinacally useful drugs, makes LasB an attractive antivirulence target for therapeutic intervention (Galdino et al., 2019). Synthesis of small-molecule inhibitors of *P. aeruginosa* LasB (*Pa*LasB) had started more than 40 years ago and since then eighteen inhibitors have been developed (Everett and Davies, 2021). The FBDD approach to develop inhibitors against *Pa*LasB started in 2012 by screening a library of 96 metal chelating fragments (CFL-1.1) (a library designed specifcally with fragments which can coordinate with the metal ion in the active site of metalloproteins) against *Pa*LasB and the initial hits were followed up by medicinal chemistry optimization program (Agrawal et al., 2008; Garner et al., 2012). This led to the development of a thiopyridone **13** (Garner et al., 2012) with an IC_50_ of 2.73 μM for *Pa*LasB but was promiscuous with regard to other mettaloproteases, and the tropolone **14** (Fullagar et al., 2013) exhibiting similar potency (IC_50_ of 1 μM for LasB) but had better selectivity over human metalloproteases, such as matrix metalloproteases (MMPs) and carbonic anhydrase II (Figure 4A). They were the first potent non-peptidic small molecule antagonists discovered (Cathcart et al., 2011) and the first targetted compounds exhibiting antiswarming activity (Garner et al., 2012).

With the aim of expanding the chemical space of *Pa*LasB inhibitors, a functional screening based on FRET-based *in vitro* assay (Nishino and Powers, 1980) was performed against *Pa*LasB using 330 fragments (Maybridge Fragment Library) and a protease inhibitor-enriched library (Baell and Holloway, 2010) comprising of 1,192 low molecular weight compounds (Kany et al., 2018a). This study led to the development of a thiobenzamide (N-(3,4-dichlorophenyl)-2-sulfanylacetamide) **15**, LasB inhibitor with thiol warhead, with an IC_50_ of 6.6 μM (Figure 4A). This was selective with regard to human MMPs. The X-ray structure of *Pa*LasB-**15** complex revealed that binding of **15** to LasB does not necessarily lead to closure of the binding site (Kany et al., 2018a), unlike that described for thermolysin-like proteases (Adekoya et al., 2015), enzymes with high structural similarity to LasB. Moreover, two molecules of **15** were observed in the structure (Figure 4B) and the binding of the second molecule was supposed to be supported by the binding of the first molecule. However, the Hill coefficient of 1 in the *in vitro* assay for **15** suggested that only one binding event was necessary for full inhibition of *Pa*LasB. Neverthless, this structure with two binding sites for **15** paved the way for the development of novel LasB inhibitors targeting the open conformation of the enzyme. Attempts to merge these two molecules into one *N*-benzylamide derivative did not provide the desired activity. Growing the non-zinc-chelating molecule of **15** in the *Pa*LasB-**15** complex led to the development of N-arylsuccinimide **16** with an IC_50_ of 4 μM, which showed, 1) significant selectivity for bacterial LasB over human MMPs and three other off-targets and 2) no signs of cytotoxicity in human cell lines (Konstantinović et al., 2020). In a another subsequent study, the zinc-chelating molecule of **15** in the *Pa*LasB-**15** complex was modified by changing the zinc-chelating moiety leading to a hydroxamate **17** (Kany et al., 2018b) with an IC_50_ of 17 μM with moderate cytotoxic effects towards mamalian cell lines. Since only one molecule of **17** was observed in the crystal structure of *Pa*LasB-**17** complex (Figure 4C), unlike that of **15** in *Pa*LasB-**15** complex, and **17** was less susceptible to oxidation in air, the authors preferred **17** over **15** for further exploration. Moreover, **17** could undergo the characteristic hinge-bending motion resulting in clusure of LasB binding site unlike open conformation as observed in *Pa*LasB-**15** complex. Overall, given their modest activities, the authors suggest that the molecules such as **15**, **16** and **17** should be considered as starting points for further chemistry rather than as potential drug development candidates which could pave the way for the rational development of selective protease inhibitors as potential new antibiotics.

Exploiting the alternative binding modes of **15** to guide efficient fragment growing resulted in a series of compounds with better activities (Kaya et al., 2021). Compound **18** thus developed had an IC_50_ of 1.2 μM (Figure 4A) and maintains the selectivity as **15**. *Pa*LasB-**18** complex structure was determined by x-ray crystallography. Here again, the binding of **18** to *Pa*LasB leads to closure of the binding site. This structure provided a deeper understanding of the possible interactions in the surrounding unoccupied space and complemented with docking analysis it paved the way for further optimization. A focused, substrate-inspired structure-based optimization of **18** (substitution of methyl group at the *para* position on the aromatic core of N-arylacetamide) resulted in compound **19** (Figure 4A) with fourteen-fold boost in activity (IC_50_ 0.48 μM) compared to **15** (173).

*In vivo* efficacy of these compounds was investigated using an insect model, *Galleria mellonella* and PA14, a virulent stain of *P. aeruginosa*. Since *Galleria mellonella* and mice show similar virulence patterns when infected with mutant PA14 starins (PA14, virulent strain of *P. aeruginosa*) this insect is likely to be a good model system to perform *in vivo* studies (Jander et al., 2000). Administering the anti-LasB compound **15** thus generated by FBDD campaign has shown significant increase in the survival of *Galleria mellonella* larvae infected with PA14. For instance, injection of 2.5 nM of 15 in the P14 infected larvae increased the survival of the larve from 43 to 73% after 65 h (Kany et al., 2018a). Compound **19** with better IC_50_ showed enhanced *in vivo* efficacy compared to **15**, thereby accelerating the translational path (Kaya et al., 2021). Antivirulent agents targeting quorum sensing of *P. aeruginosa* have previously been shown to enhance the survival of *Galleria mellonella* larvae infected with PA14 (Lu et al., 2014; Thomann et al., 2016). These compounds attain this efficacy by inhibiting the swarming and biofilm formationa rather than being bacteriocidal. For instance, presence of compound **17** reduces the formation of biofilm and release of extracellular DNA by *P. aeruginosa*. The advantage of such inhibitors is that they are capable of disrupting several important bacterial resistance mechanisms and hence open novel avenues to combat multidrug resistant strains of *P. aeruginosa*.

#### Fragment-Based Drug Discovery on PqsD

PqsD is a key enzyme in the biosynthesis of signal molecules 2-heptyl-4-hydroxyquinoline (HHQ) and *Pseudomonas* quinolone signal (PQS) that are involved in the regulation of virulence factor (pyocyanine, elastase B, lectin A, rhamnolipids, and hydrogen cyanide) production and biofilm formation in *P. aeruginosa* (Van Delden and Iglewski, 1998; Diggle et al., 2003; Déziel et al., 2005; Yang et al., 2009). It has been shown that a mutant *P. aeruginosa* having a transposon insertion in the *pqsA* gene (deficient in HHQ and PQS production) forms less biofilm than the wild type (Gallagher et al., 2002; Müsken et al., 2010). Using a ligand-based approach the first class of PqsD inhibitors were identified which repressed HHQ and PQS production and biofilm formation in *P. aeruginosa,* validating PqsD as an attractive anti-biofilm target for the development of novel anti-infectives (Storz et al., 2012). Elisabeth Weidel and co-workers performed an SPR-based fragment screening against *P. aeruginosa* PqsD (*Pa*PqsD) using a library of 500 fragments (Maybridge) with high structural diversity covering large chemical space in order to identify new scaffold for drug discovery (Koch, 2017). This screen resulted in identification of three fragments exhibiting a moderate inhibition of *Pa*PqsD (Table 3). Two compounds showed strong inhibition of *Pa*PqsD and hence these may be the starting point for future investigations.

**TABLE 3:** Fragment-like inhibitors of *Pa*PqsD obtained by a FBDD campaign with two fragments showing strong inhibition (85%) of *Pa*PqsD.

Fragment	Structure	Inhibition of *Pa*PqsD
**20**	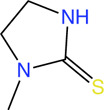	>99%
**21**	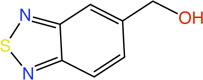	58%
**22**	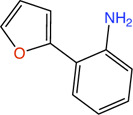	80%

### Targeting Disulfide Mediated Protein Folding

#### Fragment-Based Drug Discovery on DsbA1

**FIGURE 5:**
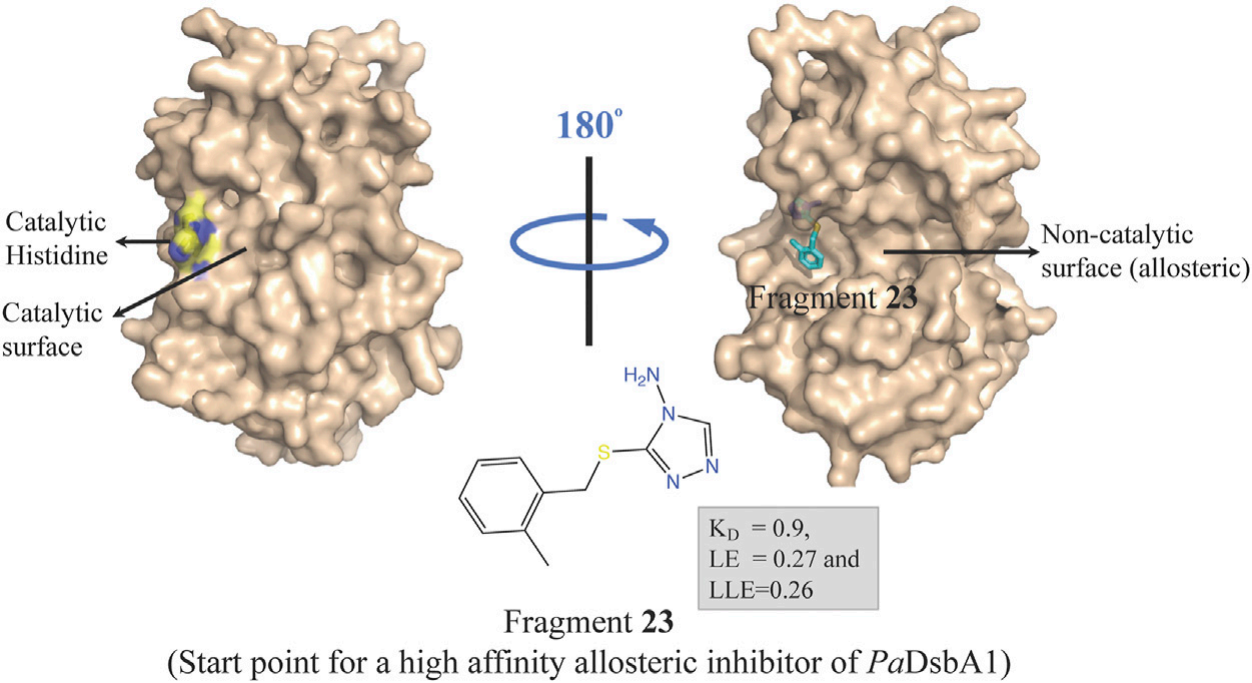
Binding site of fragment **23** (obtained by a FBDD campaign) to the non-catalytic allosteric surface site of *Pa*DsbA1 as revealed by the *Pa*DsbA1-**23** complex (pdb id 5dch) obtained using X-ray crystallography (Mohanty et al., 2017).

Oxidative protein folding is essential for assembly and function of many secreted and membrane proteins, and DSB (disulfide bond) proteins that catalyze the disulfide bond formation are essential for virulence of many Gram-negative bacteria and hence these proteins are targets for novel antibacterial drugs (Heras et al., 2009). DsbA, an enzyme of disulfide oxidoreductase family, and its membrane-bound partner DsbB together catalyze the oxidative folding of disulfide bond containing proteins, many of which are virulence factors including secreted toxins and cell surface components, such as adhesins and pili. DsbA1 in *P. aeruginosa* (*Pa*DsbA1) plays a pivotal role in the oxidative folding of virulence factors qualifying it as an attractive target for the development of new anti-virulence antimicrobials (Braun et al., 2001; Urban et al., 2001; Ha et al., 2003). In a FBDD approach, *Pa*DsbA1 was screened against a library of 1,137 fragments (Bradley et al., 2013) using ligand-detected STD NMR, which identified small molecules that bind selectively to *Pa*DsbA1 over *E. coli* DsbA (*Ec*DsbA) suggesting the feasibility of species-specific development of narrow-spectrum inhibitor of *Pa*DsbA1 (Mohanty et al., 2017). Structural characterization of the complex of *Pa*DsbA1 with fragment **23** (highest affinity fragment with K_D_ of 0.9, ligand efficiency 0.27 and lipophilic ligand efficiency of 0.26) using both X-ray crystallography and HADDOCK model revealed that the fragment is positioned at an interface between the thioredoxin (TRX) and helical domains of *Pa*DsbA1 on the non-catalytic face (Figure 5) of the enzyme. Unfortunately, fragment 1 had no inhibitory effect on the enzymatic activity of *Pa*DsbA1 as shown in an *in vitro* model-peptide folding assay. Nevertheless, these findings represent a starting point for the development of high affinity allosteric inhibitors of *Pa*DsbA1.

### Fragment-Based Drug Discovery on GRX: Development of Covelent Inhibitors of *P. aeruginosa* by Fragment-Based Drug Discovery

**FIGURE 6:**
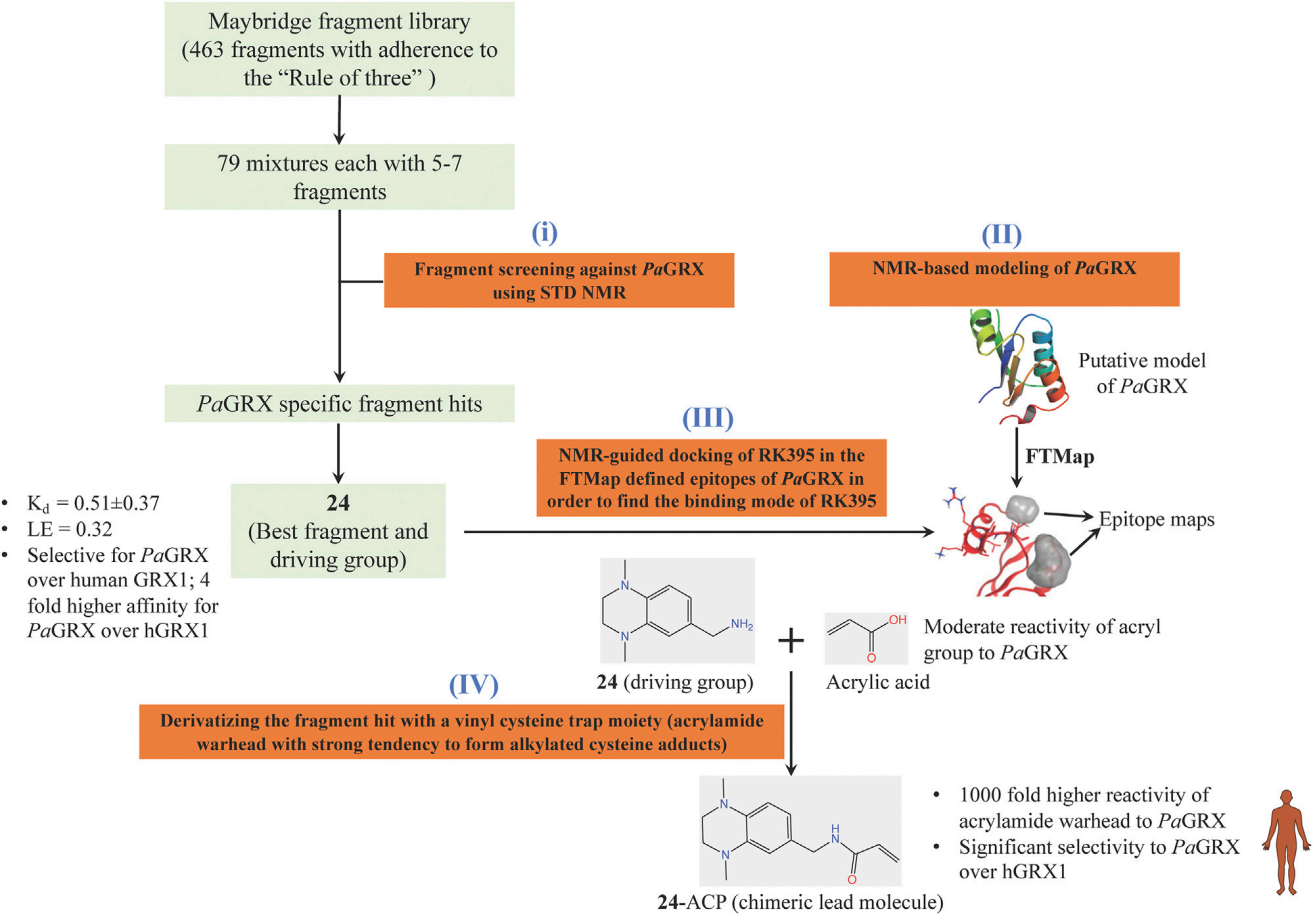
FBDD campaign using NMR against *Pa*GRX to identify best driving group, derivatization. A vinyl cysteine trap moiety resulted in the development of chimeric lead molecule with enhanced reactivity and selectivity to *Pa*GRX over human GRX1 (hGRX1). Putative model *Pa*GRX and FTMap identified epitope maps of *Pa*GRX in this figure are adopted from (Khattri et al., 2020) with permission from the authors.

Inspired by the need to develop novel classes of drug molecules targeting unconventional drug targets using a mechanism that can circumvent the efflux-mediated resistance mechanism, covalent inhibitors were sought. The present strategy involves combining a covalently reactive functional group with targeting moieties (of compounds) selective for species-specific proteins from important biochemical pathways that might have been missed due to cross-reactivity issues to the host organism. Such atypical protein targets are less likely to contain resistance-conveying mutations. Moreover, if the targeted pathway is fundamentally essential to the bacterial metabolism then infectious colonies are less likely to undergo viable mutations. Such targets from *P. aeruginosa* are the glutaredoxins. Glutaredoxins are component of thiol-disulfide glutaredoxins systems of bacteria that favour reducing conditions for the correct disulfide bonding of the functional protein and therefore they are employed by bacteria to defend against oxidative stress imposed by host (Norambuena et al., 2012). Hence glutaredoxins are considered as potential drug targets. An NMR-led FBDD campain targeted the *P. aeruginosa* glutaredoxin (*Pa*GRX) (Khattri et al., 2020). This work involved: 1) generating *Pa*GRX-specific fragment hits by screening 463 fragment molecules using independent STD NMR measurements, 2) NMR-based modeling of *Pa*GRX, 3) NMR-guided docking of hits and 4) derivatising the fragment hit with a vinyl cysteine trap moiety (acrylamide warhead with strong tendency to form alkylated cysteine adducts) to generate the chimeric lead (Figure 6). The authors show that mM to μΜ selectivity can be achieved for a few fragments against orthologous proteins and the promising fragment can be optimised to enhance the selectivity by choosing an appropriate warhead. For instance, fragment **24** which binds to *Pa*GRX with moderate specificity (K_d_ = 0.51 ± 0.37 and LE = 0.32), when coupled with acrylic acid warhead showed enhanced specificity towards the enzyme. This can be further developed to result in to thiol-transferase inhibitory drug candidate against *P. aeruginosa*.

### Targetting Effector Proteins of Type III Secretion System of *P. aeruginosa*

Type III secretion system (T3SS) plays a pivotal role in the virulence and development of antimicrobial resistance of *P. aeruginosa* by providing a diverse range of virulence factors. Readers of this review are referred to a recent review by Gertrudis Horna and Joaquim Ruiz (Horna and Ruiz, 2021b) for more details on the T3SS machinery of *P. aeruginosa*. T3SS is aimed to inject the effectors in host-cells, subverting cellular machinery and neutralising the host immune responses, thereby enhancing bacterial survival rates in a hostile environment within the macrophages. Hence, T3SS impairment opens up opportunities for developing antimicrobial agents to combat *P. aeruginosa* infections avoiding antimicrobial pressure on this and other microorganisms (Aburto-Rodríguez et al., 2021; Horna and Ruiz, 2021a). Until now six effector proteins (ExoS, ExoT, ExoU, ExoY, PemA, PemB) have been reported to be encoded by the T3SS of *P. aeruginosa* (Hueck, 1998; Barbieri and Sun, 2004; Hauser, 2009; Burstein et al., 2015), while four new putative effectors have recently been proposed (Zelikman et al., 2020). Of these, ExoU, ExoS, ExoT and ExoY have been studied most extensively. Drug development other than FBDD campaign have produced small molecule inhibitors of ExoU (Lee et al., 2007; Foulkes et al., 2021), ExoS (Arnoldo et al., 2008) and an inhibitor leading to decrease in secretion of ExoT and ExoY (Sheremet et al., 2018). Most of the components are yet to be investigated for inhibitor development. Thus, there is scope for the development of small molecule inhibitors using FBDD targeting these and other unexplored effectors which could interfere with these effectors and enhance the internalisation of *P. aeruginosa* infections by macrophages.

## Fragment-Based Drug Discovery Against *P. aeruginosa* at University of Cambridge

In 2017, United Kingdom Cystic Fibrosis Trust announced a £10 million research partnership with the University of Cambridge to create the first United Kingdom Cystic Fibrosis Innovation Hub with an aim to develop life-changing new treatments for people with CF. The CF innovation hub is hosted by Department of Medicine and led by Professor Andres Floto. It harnesses a multidisciplinary approach with the aim of delivering new treatments for bacterial infections, chronic inflammation, and lung repair for patients with CF. The Arif and Blundell contribution to the United Kingdom Cystic Fibrosis Innovation Hub at the University of Cambridge is to develop new antibiotics against *P. aeruginosa* and *M. abscessus*. We use computational approaches to understand the gene products that might be appropriate targets by modelling three-dimensional structures (Šali and Blundell, 1993), often of multiprotein assemblies, understanding the impacts of mutations (Pires et al., 2014; Pandurangan et al., 2017; Pandurangan and Blundell, 2020) and assessing the essentiality of the gene. We then use structure-guided fragment-based drug discovery, pioneered in Cambridge in Astex in 1999 (see review Blundell et al., 2002) (Blundell et al., 2002), to develop candidate molecules in our biochemistry and structural biology laboratories and in collaboration with medicinal chemists in the group founded by Professor Chris Abell in Cambridge. Arif and Blundell also work very closely with Prof. Andres Floto, Research Director of the Cambridge Centre for Lung Infection at Papworth Hospital, Cambridge. His team carries out *in vivo* biological assays to test for the effects of elaborated compounds (inhibitors) on the cell system.

**FIGURE 7:**
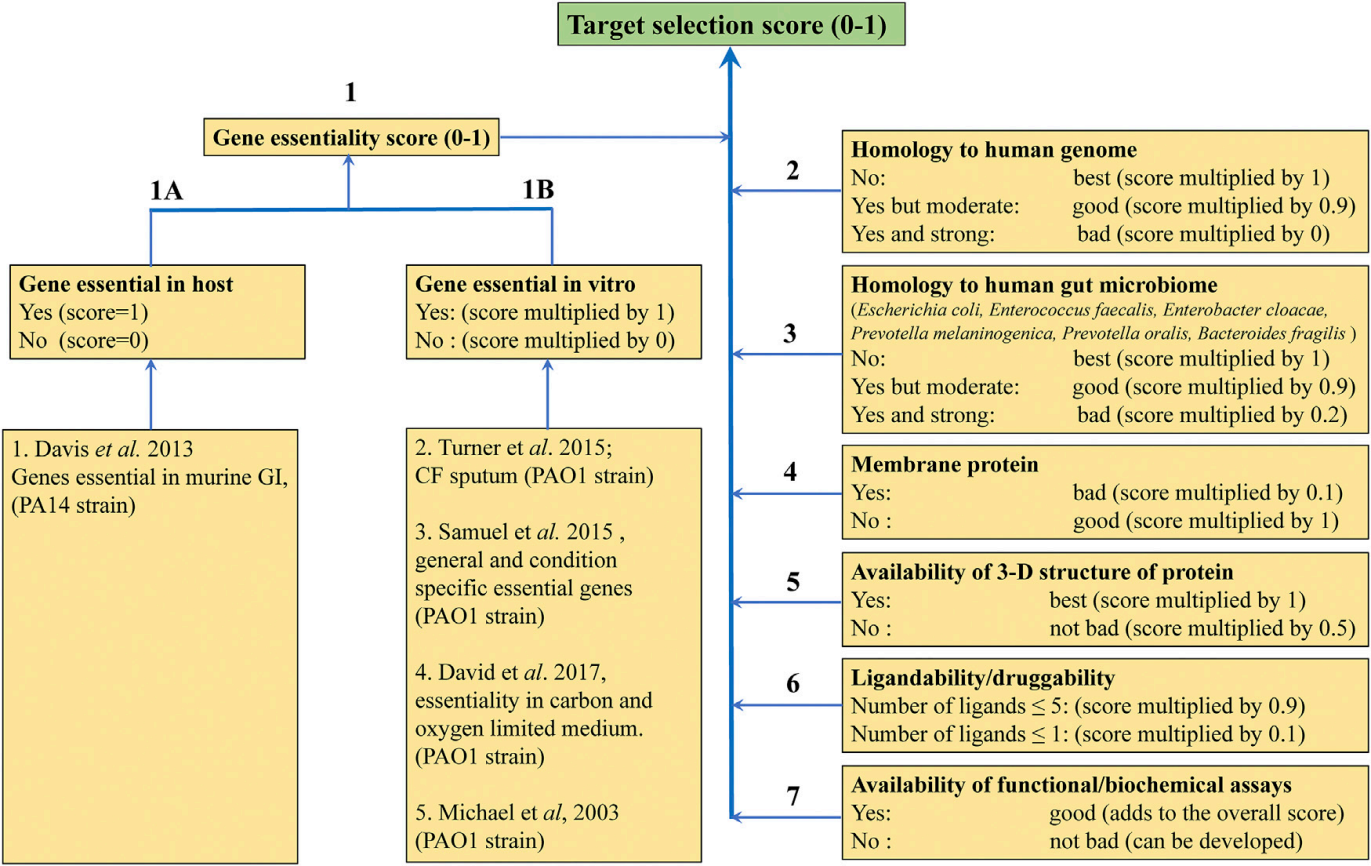
Criteria for selecting protein targets from *P. aeruginosa* to be studied using FBDD. Different weights were given to the different criteria according to their impact on target selection.

Funded as part of the Cystic Fibrosis Hub we began to focus on targets from *P. aeruginosa and M. abscessus*. A number of considerations for target selection such as gene essentiality *in vitro* and *in vivo* (Liberati et al., 2006; Skurnik et al., 2013; Lee et al., 2015; Turner et al., 2015; Basta et al., 2017), for example having little or no sequence identity to human or gut microbiome counterparts, the availability of apo and liganded crystal structures that suggest good ligandability, cellular localization of the protein, feasibility of functional/enzymatic assays for the target, were applied to prepare a list of targets from *P. aeruginosa* with ranking based on associated target selection scores (Figure 7).

### Targeting DapD: An Essential Protein of the Lysine Biosynthesis Pathway

**FIGURE 8:**
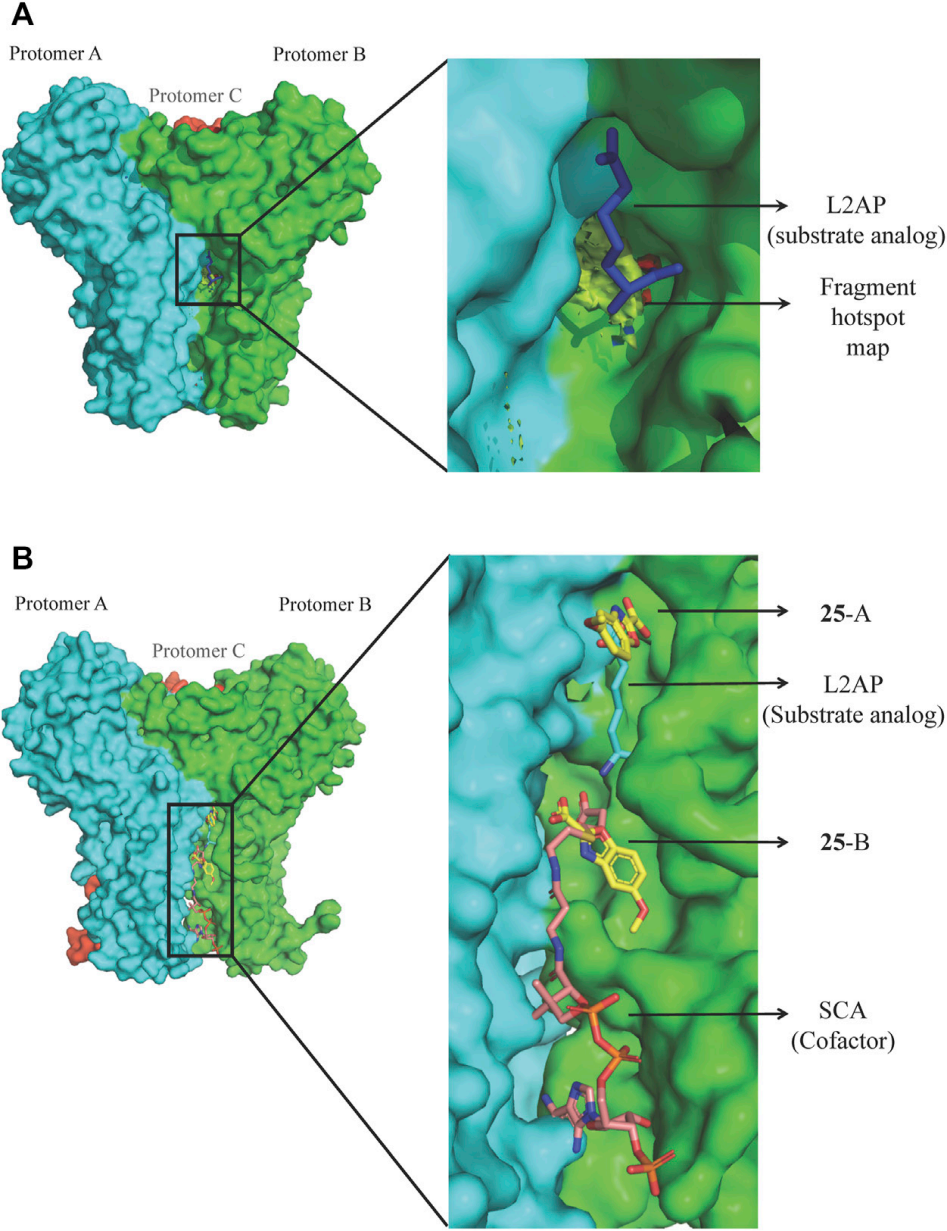
FBDD against *Pa*DapD. **(A)** Binding site and fragment hotspot maps of *Pa*DapD. The yellow region in the zoomed view represents the hydrophobic map while hydrogen-bond acceptor and hydrogen-bond donors are represented in red and blue, respectively. **(B)** ligands from various *Pa*DapD-ligand complexes mapped on the structure of *Pa*DapD-**25** complex. **25** binds at two different sites in the protomer-protomer interfaces of *Pa*DapD. **25** at site A overlaps with the binding site of the substrate analog L2AP, while that at site B overlaps with the binding site of the cofactor SCA.

Bacterial genetic studies have suggested that the lysine biosynthesis pathway is essential and offers several potential antibacterial enzyme targets that could be explored (Cox et al., 2000; Hutton et al., 2007; Gillner et al., 2013). DapD (2,3,4,5-tetrahydropyridine-2,6-dicarboxylate *N*-succinyltransferase), a product of an essential gene involved in the lysine biosynthesis pathway, was selected as an initial target. *P. aeruginosa* DapD (*Pa*DapD) was ranked at the very top in the list of potential targets. The lack of human homolog of DapD and maximum identity of 36% to a homolog from human gut microbiome qualifies it for a potential target as this would likely avoid any cross reactivity of the developed inhibitors. DapD catalyses the conversion of cyclic tetrahydrodipicolinate (THDP) into the acyclic *N*-succinyl-*L*-2-amino-6-oxopimelate using succinyl-CoA (SCA) as cofactor. Detailed structural analyses of the available complexes with substrate and cofactors suggest that a long narrow crevice is formed at each interface of the *Pa*DapD trimer where the reactants bind (Schnell et al., 2012). Analysis using the HOTSPOT server (50) strongly suggests that this binding site is suitable for the binding of fragments (Figure 8A). We speculated that elaborated small molecules that bind to these sites will potentially block the binding of substrate/cofactor and will eventually inactivate the enzyme.

**FIGURE 9:**
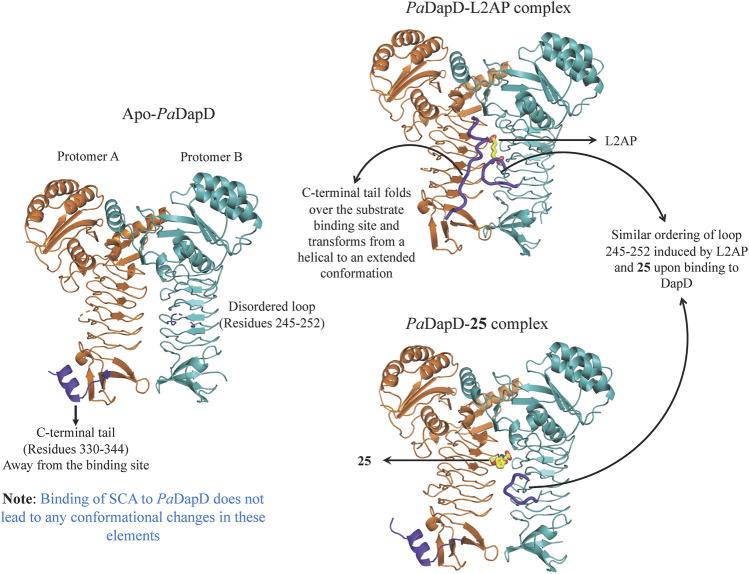
Conformational changes observed in *Pa*DapD upon binding of ligands. Significant changes are observed in the C-terminal tail 330–344 and loop 245–252 (both in purple) upon binding of the substrate analog L2AP alone to *Pa*DapD. Interestingly, binding of **25** leads to ordering of loop 245–252 in a similar way to that observed on binding of substrate analog L2AP to *Pa*DapD.

An initial fragment screening employing the differential scanning fluorimetry (DSF) using our in-house library of 960 fragments resulted in 10 promising compounds that could bind to the protein, as all led to a positive shift in the thermal melt temperature of the protein. We then produced crystal structures of apo-*Pa*DapD and binary complexes of *Pa*DapD with fragment **25**, the cofactor succinyl CoA (SCA); with the substrate analogue L-2-amino pimelic acid (L2AP). SCA binds in the narrow pocket at the interface formed between the N-terminal, middle and C-terminal part of the neighbouring protomers. L2AP binds at the N-terminal region of the pocket near succinyl β-mercaptoethylamine moiety of SCA (Figure 8B). **25** binds at a location (**25**-A) coinciding with the L2AP binding site. At higher concentrations of **25** a second binding site (**25**-B) is observed that overlaps with part of the SCA binding site (Figure 8B). The structure of *Pa*DapD-L2AP complex differs from the apo-*Pa*DapD complex in having the loop (residues 245–252) ordered and interacting with the ligand (Figure 9). Another large change is observed in the C-terminal tail region, which, unlike in the apo-*Pa*DapD and *Pa*DapD-SCA complexes where it was helical, is extended and covers the active site resulting in a cage like structure of the binding pocket (Figure 9). A comparison of the binary complexes of L2AP and **25** demonstrates a similarity in the conformational changes observed upon their binding to the active site (Figure 9), which might guide the design of inhibitors against the enzyme.

**FIGURE 10:**
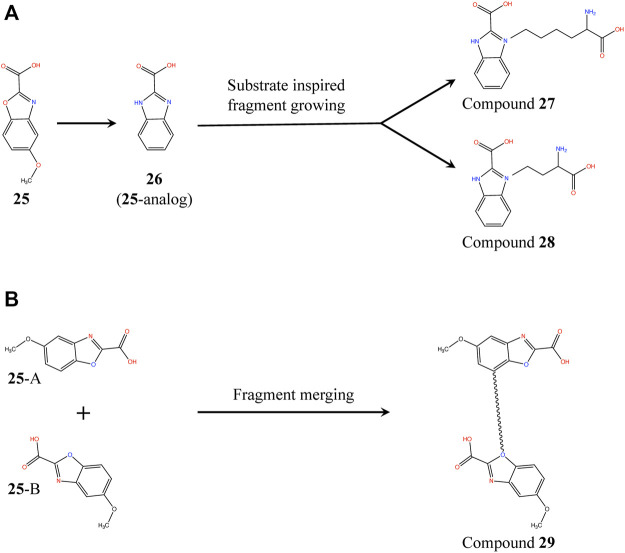
Fragment growing and fragment merging strategies based on the structure of *Pa*DapD-**25** and *Pa*DapD-L2AP complexes. **(A)** substrate-inspired growing of an **25**-analog (**26**) based on the structures of *Pa*DapD-**26** and *Pa*DapD-L2AP complexes resulting in compound **27** and **28**. The feasibility of the fragment growing is enhanced when performed on **26**, **(B)** Merging of **25** at sites A and B of *Pa*DapD employing suitable linker based on the structure of *Pa*DapD-**25** complex to develop compound **29**. The binding and inhibition of these compounds to *Pa*DapD needs to be investigated.

Proximity of **25** and substrate analog L2AP suggests a structure-guided substrate-inspired growing of the fragment (Figure 10A) in order to potentiate it for binding to *Pa*DapD. Another strategy could be to merge the fragments on the basis of the two binding sites observed in the *Pa*DapD-**25** complex structure using an appropriate length of linker (Figure 10B). The former could be achieved by first synthesizing an analog (**26**) to which substituents could be chemically added. We had planned to perform the syntheses with the help of our chemist collaborator (Dr J. Mayol-Llinas and Prof. Chris Abell) but these have been interrupted by the untimely death of Professor Chris Abell. The plan remains that the resulting compounds (compounds **27**, **28** and **29**) will then be tested for the binding to *Pa*DapD using DSF, ITC and SPR.

### Targeting KdsA: An Enzyme in Lipopolysaccharide Biosynthesis Pathway

As LPS has an important role in the structural integrity of the bacterium and its defence against the host, the enzymes of the LPS biosynthesis pathway are attractive drug targets. Four of them KdsA, KdsB, LpxC and WaaG were selected for homology modeling with the aim of developing inhibitors against them. Subsequently, the essentiality of KdsA for the survival and virulence of the *P. aeruginosa* was validated experimentally (Perumal et al., 2011).

**FIGURE 11:**
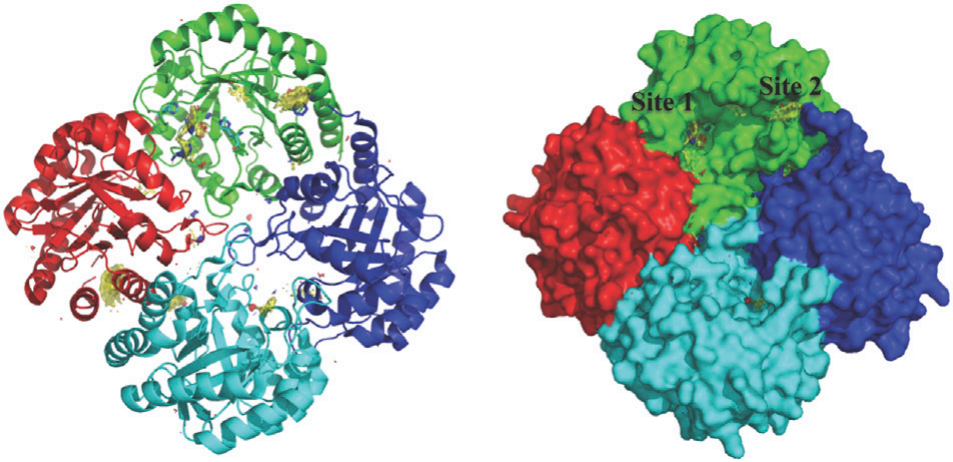
Tetrameric structure of PaKdsA and binding sites/hotspots identified by HOTSPOT. Two sites, site A and B have been identified to which fragments are predicted to bind.

KdsA, a 3-deoxy-D-manno-octulosonate 8-phosphate (KDO8P) synthase, catalyses the condensation reaction between D-arabinose 5-phosphate (A5P) and phosphoenolpyruvate (PEP). This enzymatic reaction plays an essential role in the synthesis and assembly process of lipopolysaccharides of most Gram-negative bacteria and is therefore an attractive target for the design of novel antibacterial drugs. The crystal structures of *E. coli* KdsA as binary complexes with the substrate, PEP, and with a mechanism-based inhibitor (K_d_ = 0.4 µM), gave insight about its mechanism of action and inhibition. Interestingly, KdsA belongs to a family of PEP-utilizing enzymes, two of which, UDPGlcNAc enolpyruvoyl transferase (MurZ) and 5-enolpyruvoylshikimate-3-phosphate synthase (EPSPS), are targeted by the antibiotic Fosfomycin and by the herbicide glyphosate, respectively. The crystal structure of *P. aeruginosa* KdsA (*Pa*KdsA) in its binary complex with phosphoenolpyruvate (PEP) was reported in the year 2013 (Nelson et al., 2013). *Pa*KdsA is a tetrameric enzyme and the binding site/hotspot of the *P. aeruginosa* KdsA was probed by generating fragment hotspot maps using the *HOTSPOT* program (50), which suggested the presence of promising pocket for targeting new therapeutics (Figure 11).

A fragment library of 480 non-redundant fragments was used for screening against *Pa*KdsA employing DSF. 56 fragments showed negative shift in melting temperature while 14 fragments showed positive delta Tm shift. Unfortunately, none of them showed any binding to *Pa*KdsA as determined using ITC and X-ray crystallography.

The protein was also used to produce crystals of apo protein for a crystal-based fragment screening, and in complex with the substrates, PEP and A5P, in order to locate the active site which could be used for a virtual screening campaign. The attempt with PEP resulted in *Pa*KdsA-PEP complex and in the presence of A5P resulted in PaKdsA-A5P complex. When the two substrates were mixed and used for cocrystallization, a product (KDO8P) bound crystal, *Pa*KdsA-KDO8P, was obtained. PEP binds deep in the binding pocket and A5P binds above PEP in a more solvent exposed location. KDO8P in its complex with *Pa*KdsA spans the location of both PEP and A5P.

**FIGURE 12:**
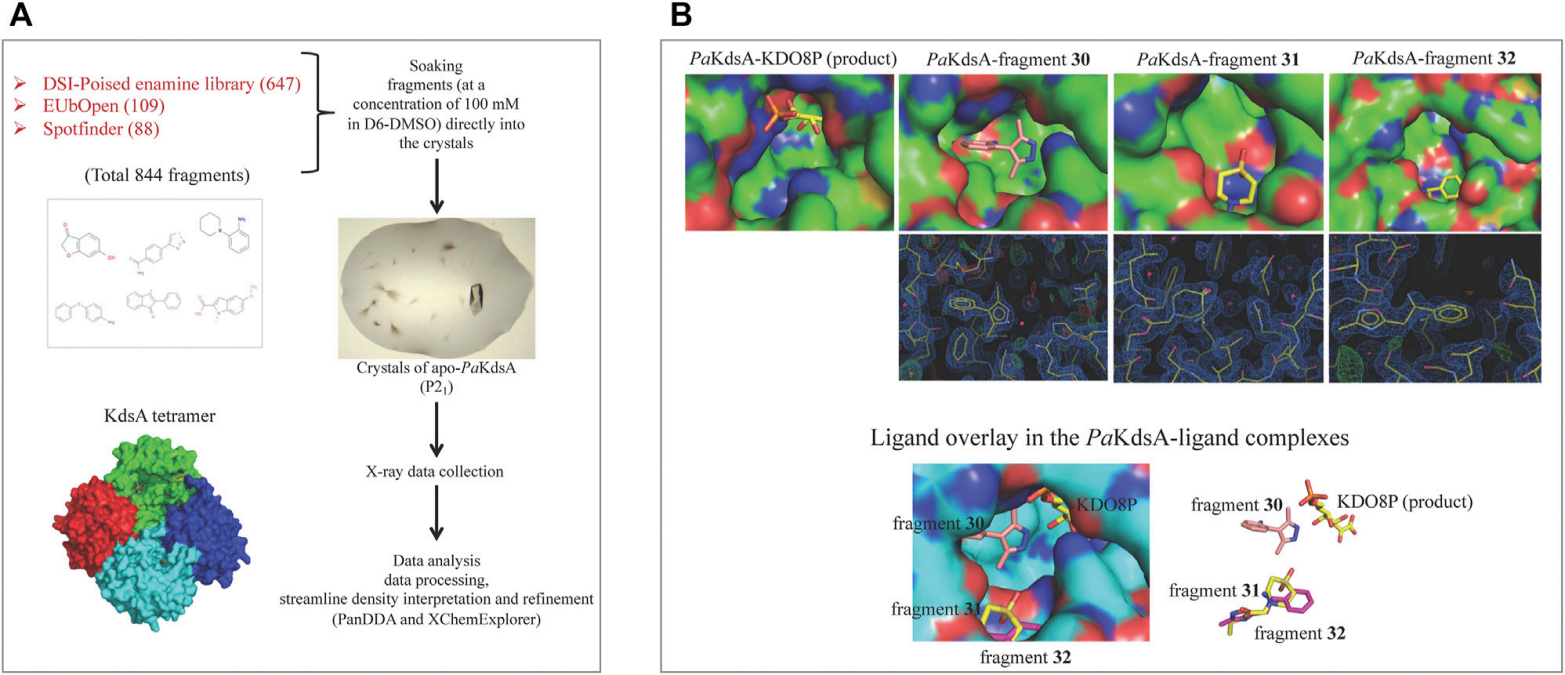
FBDD campaign against *Pa*KdsA **(A)** Crystal-based fragment screening against *Pa*KdsA at the XChem facility of the Diamond Light Source. **(B)** Fragment hits (**30**, **31** and **32**) obtained from the screening. The top panel shows location of fragments and the product KDO8P in their respective complexes with *Pa*KdsA. The middle panel shows the electron density maps of fragments and the bottom panel shows the fragments from their complexes to *Pa*KdsA mapped on the *Pa*KdsA-KDO8P complex in order to compare their locations in the active site of *Pa*KdsA.

A high-throughput crystal-based fragment screening against *Pa*KdsA was carried out at the XChem facility of Diamond Light Source (Figure 12A) using a total of 886 crystals, 42 for solvent characterisation and 844 for soaking fragments from three different fragment libraries, namely, DSI poised (Enamine) (647 fragments), EUbOpen (109 fragments) and Spotfinder (88 fragments). 17 hits were obtained after this screening, most of the fragments were bound at the sites generated by the crystal lattice while three fragments (**30**, **31** and **32**) were bound near the active site of the enzyme (Figure 12B). The fragments binding near the active site could be starting points for hit-to-lead compound development against *Pa*KdsA.

## Conclusion

Our review of structure-guided fragment-based drug discovery to target *P. aeruginosa* infections demonstrates its great potential in the design of new medicines to combat infections in the lungs of cystic fibrosis patients. However, although *P. aeruginosa* is a common strict aerobic bacterium that can cause disease in plants and animals, including humans, research towards the development of molecules to target it, using fragment-based approaches, remains at an early stage. Nevertheless, as our review demonstrates, the studies are encouraging, although small in number. In the future, further investment will be required in taking leads though the clinic and this is as always a challenge for genetic diseases, which affect a small percentage of the population, even those that are relatively common in wealthy Western nations of North America and Western Europe. The challenge is exacerbated by the fact that this ubiquitous multidrug-resistant pathogen, *P. aeruginosa*, exhibits advanced antibiotic resistance mechanisms. We remain grateful to organisations such as the Cystic Fibrosis Trust that are giving generous support to research groups such as ours in moving new candidates into the clinic.

## References

[B1] Aburto-Rodríguez N. A. Muñoz-Cázares N. Castro-Torres V. A. González-Pedrajo B. Díaz-Guerrero M. García-Contreras R. (2021). Anti-pathogenic Properties of the Combination of a T3ss Inhibitory Halogenated Pyrrolidone with C-30 Furanone. Molecules 26, 7635. 10.3390/molecules26247635 34946717 PMC8707098

[B2] Adams P. D. Afonine P. V. Bunkóczi G. Chen V. B. Davis I. W. Echols N. (2010). PHENIX: A Comprehensive Python-Based System for Macromolecular Structure Solution. Acta Crystallogr. D Biol. Cryst. 66, 213–221. 10.1107/S0907444909052925 20124702 PMC2815670

[B3] Adekoya O. A. Sjøli S. Wuxiuer Y. Bilto I. Marques S. M. Santos M. A. (2015). Inhibition of Pseudolysin and Thermolysin by Hydroxamate-Based MMP Inhibitors. Eur. J. Med. Chem. 89, 340–348. 10.1016/j.ejmech.2014.10.009 25462250

[B60] Agrawal A. Johnson S. L. Jacobsen J. A. Miller M. T. Chen L-H. Pellecchia M. (2010). Chelator Fragment Libraries for Targeting Metalloproteinases. Available at: 10.1002/cmdc.200900516 PMC2825879 20058293

[B4] Anantharajah A. Mingeot-Leclercq M.-P. Van Bambeke F. (2016). Targeting the Type Three Secretion System in *Pseudomonas aeruginosa* . Trends Pharmacol. Sci. 37, 734–749. 10.1016/j.tips.2016.05.011 27344210

[B5] Anderson M. S. Bull H. G. Galloway S. M. Kelly T. M. Mohan S. Radika K. (199319865). UDP-N-acetylglucosamine Acyltransferase of *Escherichia coli*. The First Step of Endotoxin Biosynthesis Is Thermodynamically Unfavorable. J. Biol. Chem. 268, 19858–19865. 10.1016/s0021-9258(19)36592-5 8366124

[B6] Arnoldo A. Curak J. Kittanakom S. Chevelev I. Lee V. T. Sahebol-Amri M. (2008). Identification of Small Molecule Inhibitors of *Pseudomonas aeruginosa* Exoenzyme S Using a Yeast Phenotypic Screen. Plos Genet. 4, e1000005. 10.1371/journal.pgen.1000005 18454192 PMC2265467

[B7] Asmari M. Ratih R. Alhazmi H. A. El Deeb S. (2018). Thermophoresis for Characterizing Biomolecular Interaction. Methods 146, 107–119. 10.1016/j.ymeth.2018.02.003 29438829

[B8] Baek M. DiMaio F. Anishchenko I. Dauparas J. Ovchinnikov S. Lee G. R. (2021). Accurate Prediction of Protein Structures and Interactions Using a Three-Track Neural Network. Science 373, 871–876. 10.1126/science.abj8754 34282049 PMC7612213

[B9] Baell J. B. Holloway G. A. (2010). New Substructure Filters for Removal of pan Assay Interference Compounds (PAINS) from Screening Libraries and for Their Exclusion in Bioassays. J. Med. Chem. 53, 2719–2740. 10.1021/jm901137j 20131845

[B10] Bancet A. Raingeval C. Lomberget T. Le Borgne M. Guichou J.-F. Krimm I. (2020). Fragment Linking Strategies for Structure-Based Drug Design. J. Med. Chem. 63, 11420–11435. 10.1021/acs.jmedchem.0c00242 32539387

[B11] Barbieri J. T. Sun J. (2004). *Pseudomonas aeruginosa* ExoS and ExoT. Rev. Physiol. Biochem. Pharmacol. 152, 79–92. 10.1007/s10254-004-0031-7 15375697

[B12] Bartha I. Di Iulio J. Venter J. C. Telenti A. (2018). Human Gene Essentiality. Nat. Rev. Genet. 19, 51–62. 10.1038/nrg.2017.75 29082913

[B13] Bassetti M. Vena A. Croxatto A. Righi E. Guery B. (2018). How to Manage *Pseudomonas aeruginosa* Infections. Dic 7, 1–18. 10.7573/dic.212527 PMC5978525 29872449

[B14] Basta D. W. Bergkessel M. Newman D. K. (2017). Identification of Fitness Determinants during Energy-Limited Growth Arrest in *Pseudomonas aeruginosa* . MBio 8, 1–17. 10.1128/mBio.01170-17 PMC5705914 29184024

[B15] Baurin N. Aboul-Ela F. Barril X. Davis B. Drysdale M. Dymock B. (2004). Design and Characterization of Libraries of Molecular Fragments for Use in NMR Screening against Protein Targets. J. Chem. Inf. Comput. Sci. 44, 2157–2166. 10.1021/ci049806z 15554686

[B16] Bielska E. Lucas X. Czerwoniec A. M. Kasprzak J. H. Kaminska K. M. Bujnicki J. (2011). Virtual Screening Strategies in Drug Design - Methods and Applications. bta 3, 249–264. 10.5114/bta.2011.46542

[B17] Blair H. A. (2021). Sotorasib: First Approval. Drugs 81, 1573–1579. 10.1007/s40265-021-01574-2 34357500 PMC8531079

[B18] Blundell T. L. Jhoti H. Abell C. (2002). High-throughput Crystallography for lead Discovery in Drug Design. Nat. Rev. Drug Discov. 1, 45–54. 10.1038/nrd706 12119609

[B19] Boehm H.-J. Boehringer M. Bur D. Gmuender H. Huber W. Klaus W. (2000). Novel Inhibitors of DNA Gyrase: 3D Structure Based Biased Needle Screening, Hit Validation by Biophysical Methods, and 3D Guided Optimization. A Promising Alternative to Random Screening. J. Med. Chem. 43, 2664–2674. 10.1021/jm000017s 10893304

[B20] Bollag G. Tsai J. Zhang J. Zhang C. Ibrahim P. Nolop K. (2012). Vemurafenib: the First Drug Approved for BRAF-Mutant Cancer. Nat. Rev. Drug Discov. 11, 873–886. 10.1038/nrd3847 23060265

[B21] Boulant T. Boudehen Y.-M. Filloux A. Plesiat P. Naas T. Dortet L. (2018). Higher Prevalence of PldA, a *Pseudomonas aeruginosa* Trans-kingdom H2-type VI Secretion System Effector, in Clinical Isolates Responsible for Acute Infections and in Multidrug Resistant Strains. Front. Microbiol. 9, 1–7. 10.3389/fmicb.2018.02578 30420847 PMC6215852

[B22] Bradley C. D. Craig J. M. Jamie S. S. Martin J. S. (2013). Design and Evaluation of the Performance of an NMR Screening Fragment Library. Aust. J. Chem. 66, 1465–1472.

[B23] Braun P. Ockhuijsen C. Eppens E. Koster M. Bitter W. Tommassen J. (2001). Maturation of *Pseudomonas aeruginosa* Elastase. J. Biol. Chem. 276, 26030–26035. 10.1074/jbc.M007122200 11350952

[B24] Burgess R. R. (2018). A Brief Practical Review of Size Exclusion Chromatography: Rules of Thumb, Limitations, and Troubleshooting. Protein Expr. Purif. 150, 81–85. 10.1016/j.pep.2018.05.007 29777748

[B25] Burstein D. Satanower S. Simovitch M. Belnik Y. Zehavi M. Yerushalmi G. (2015). Novel Type III Effectors in *Pseudomonas aeruginosa* . MBio 6, 3–8. 10.1128/mBio.00161-15 PMC4453518 25784698

[B26] Campbell J. W. Cronan J. E. (2001). Bacterial Fatty Acid Biosynthesis: Targets for Antibacterial Drug Discovery. Annu. Rev. Microbiol. 55, 305–332. 10.1146/annurev.micro.55.1.305 11544358

[B27] Capelli D. Parravicini C. Pochetti G. Montanari R. Temporini C. Rabuffetti M. (2020). Surface Plasmon Resonance as a Tool for Ligand Binding Investigation of Engineered GPR17 Receptor, a G Protein Coupled Receptor Involved in Myelination. Front. Chem. 7, 1–14. 10.3389/fchem.2019.00910 PMC6966494 31998697

[B28] Cathcart G. R. A. Quinn D. Greer B. Harriott P. Lynas J. F. Gilmore B. F. (2011). Novel Inhibitors of the *Pseudomonas aeruginosa* Virulence Factor LasB: A Potential Therapeutic Approach for the Attenuation of Virulence Mechanisms in Pseudomonal Infection. Antimicrob. Agents Chemother. 55, 2670–2678. 10.1128/AAC.00776-10 21444693 PMC3101427

[B29] Chan D. I. Vogel H. J. (2010). Current Understanding of Fatty Acid Biosynthesis and the Acyl Carrier Protein. Biochem. J. 430, 1–19. 10.1042/BJ20100462 20662770

[B30] Chao M. C. Abel S. Davis B. M. Waldor M. K. (2016). The Design and Analysis of Transposon Insertion Sequencing Experiments. Nat. Rev. Microbiol. 14, 119–128. 10.1038/nrmicro.2015.7 26775926 PMC5099075

[B31] Chavanieu A. Pugnière M. (2016). Developments in SPR Fragment Screening. Expert Opin. Drug Discov. 11, 489–499. 10.1517/17460441.2016.1160888 26948323

[B32] Chen A. Y. Adamek R. N. Dick B. L. Credille C. V. Morrison C. N. Cohen S. M. (2019). Targeting Metalloenzymes for Therapeutic Intervention. Chem. Rev. 119, 1323–1455. 10.1021/acs.chemrev.8b00201 30192523 PMC6405328

[B33] Chen H. Engkvist O. Wang Y. Olivecrona M. Blaschke T. (2018). The Rise of Deep Learning in Drug Discovery. Drug Discov. TodayToday 23, 1241–1250. 10.1016/j.drudis.2018.01.039 29366762

[B34] Chen I.-J. Hubbard R. E. (2009). Lessons for Fragment Library Design: Analysis of Output from Multiple Screening Campaigns. J. Comput. Aided. Mol. Des. 23, 603–620. 10.1007/s10822-009-9280-5 19495994

[B35] Cohen F. Aggen J. B. Andrews L. D. Assar Z. Boggs J. Choi T. (2019). Optimization of LpxC Inhibitors for Antibacterial Activity and Cardiovascular Safety. ChemMedChem 14, 1560–1572. 10.1002/cmdc.201900287 31283109

[B36] Cohen S. N. Chang A. C. Y. Boyer H. W. Helling R. B. (1973). Construction of Biologically Functional Bacterial Plasmids *In Vitro* . Proc. Natl. Acad. Sci. 70, 3240–3244. 10.1073/pnas.70.11.3240 4594039 PMC427208

[B37] Collins P. M. Ng J. T. Talon R. Nekrosiute K. Krojer T. Douangamath A. (2017). Gentle, Fast and Effective crystal Soaking by Acoustic Dispensing. Acta Cryst. Sect D Struct. Biol. 73, 246–255. 10.1107/S205979831700331X 28291760 PMC5349437

[B38] Cox R. J. Sutherland A. Vederas J. C. (2000). Bacterial Diaminopimelate Metabolism as a Target for Antibiotic Design. Bioorg. Med. Chem. 8, 843–871. 10.1016/S0968-0896(00)00044-4 10881998

[B39] Crull M. R. Somayaji R. Ramos K. J. Caldwell E. Mayer-Hamblett N. Aitken M. L. (2018). Changing Rates of Chronic *Pseudomonas aeruginosa* Infections in Cystic Fibrosis: A Population-Based Cohort Study. Clin. Infect. Dis. 67, 1089–1095. 10.1093/cid/ciy215 29534149 PMC6137120

[B40] Cryz S. J. Pitt T. L. Fürer E. Germanier R. (1984). Role of Lipopolysaccharide in Virulence of *Pseudomonas aeruginosa* . Infect. Immun. 44, 508–513. 10.1128/iai.44.2.508-513.1984 6425224 PMC263549

[B41] Cukier C. D. Hope A. G. Elamin A. A. Moynie L. Schnell R. Schach S. (2013). Discovery of an Allosteric Inhibitor Binding Site in 3-Oxo-Acyl-ACP Reductase from *Pseudomonas aeruginosa* . ACS Chem. Biol. 8, 2518–2527. 10.1021/cb4005063 24015914 PMC3833349

[B42] Cystic Fibrosis Foundation 2020 Annual Data Report (2021). Cystic Fibrosis Foundation 2020 Annual Data Report.

[B43] Cystic Fibrosis Foundation (2017). Cystic Fibrosis Foundation Patient Registry. 2016 Annual Data Report. Bethesda, MD: Cystic Fibrosis Foundation.

[B44] Dalvit C. Fogliatto G. Stewart A. Veronesi M. Stockman B. (2001). WaterLOGSY as a Method for Primary NMR Screening: Practical Aspects and Range of Applicability. J. Biomol. NMR 21, 349–359. 10.1023/A:1013302231549 11824754

[B46] Dangkulwanich M. Raetz C. R. H. Williams A. H. (2019). Structure Guided Design of an Antibacterial Peptide that Targets UDP-N-Acetylglucosamine Acyltransferase. Sci. Rep. 9, 1–10. 10.1038/s41598-019-40418-8 30850651 PMC6408518

[B47] Davis P. B. (2006). Cystic Fibrosis since 1938. Am. J. Respir. Crit. Care Med. 173, 475–482. 10.1164/rccm.200505-840OE 16126935

[B48] de Souza Neto L. R. Moreira-Filho J. T. Neves B. J. Maidana R. L. B. R. Guimarães A. C. R. Furnham N. (2020). In Silico Strategies to Support Fragment-To-Lead Optimization in Drug Discovery. Front. Chem. 8, 1–18. 10.3389/fchem.2020.00093 32133344 PMC7040036

[B49] Déziel E. Gopalan S. Tampakaki A. P. Lépine F. Padfield K. E. Saucier M. (2005). The Contribution of MvfR to *Pseudomonas aeruginosa* Pathogenesis and Quorum Sensing Circuitry Regulation: Multiple Quorum Sensing-Regulated Genes Are Modulated without Affecting lasRI, rhlRI or the Production of N-Acyl- L-Homoserine Lactones. Mol. Microbiol. 55, 998–1014. 10.1111/j.1365-2958.2004.04448.x 15686549

[B50] Diggle S. P. Winzer K. Chhabra S. R. Worrall K. E. Cámara M. Williams P. (2003). The *Pseudomonas aeruginosa* Quinolone Signal Molecule Overcomes the Cell Density-Dependency of the Quorum Sensing Hierarchy, Regulates Rhl-dependent Genes at the Onset of Stationary Phase and Can Be Produced in the Absence of LasR. Mol. Microbiol. 50, 29–43. 10.1046/j.1365-2958.2003.03672.x 14507361

[B65] Douangamath A. Powell A. Fearon D. Collins P. M. Talon R. Krojer T. (2020). Achieving Efficient Fragment Screening at XChem Facility at Diamond Light Source Published. 10.3791/62414 34125095

[B51] Dundas J. Ouyang Z. Tseng J. Binkowski A. Turpaz Y. Liang J. (2006). CASTp: Computed Atlas of Surface Topography of Proteins with Structural and Topographical Mapping of Functionally Annotated Residues. Nucleic Acids Res. 34, W116–W118. 10.1093/nar/gkl282 16844972 PMC1538779

[B52] Edelsbrunner H. Mücke E. P. (1994). Three-Dimensional Alpha Shapes. ACM Trans. Graph. 13, 43–72. 10.1145/174462.156635

[B53] El Zowalaty M. E. Al Thani A. A. Webster T. J. El Zowalaty A. E. Schweizer H. P. Nasrallah G. K. (2015). *Pseudomonas aeruginosa*: Arsenal of Resistance Mechanisms, Decades of Changing Resistance Profiles, and Future Antimicrobial Therapies. Future Microbiol. 10, 1683–1706. 10.2217/fmb.15.48 26439366

[B54] Elborn J. S. (2016). Cystic Fibrosis. The Lancet 388, 2519–2531. 10.1016/S0140-6736(16)00576-6 27140670

[B55] Emsley P. Lohkamp B. Scott W. G. Cowtan K. (2010). Features and Development ofCoot. Acta Crystallogr. D Biol. Cryst. 66, 486–501. 10.1107/S0907444910007493 20383002 PMC2852313

[B56] Erlanson D. A. Fesik S. W. Hubbard R. E. Jahnke W. Jhoti H. (2016). Twenty Years on: The Impact of Fragments on Drug Discovery. Nat. Rev. Drug Discov. 15, 605–619. 10.1038/nrd.2016.109 27417849

[B45] Erlanson D. (2021). Fragments in the Clinic. Availableat: https://practicalfragments.blogspot.com/2021/11/fragments-in-clinic-2021-edition.html .

[B57] Erwin A. L. (2016). Antibacterial Drug Discovery Targeting the Lipopolysaccharide Biosynthetic Enzyme LpxC. Cold Spring Harb. Perspect. Med. 6, a025304. 10.1101/cshperspect.a025304 27235477 PMC4930914

[B58] Eşkazan A. E. (2021). Asciminib in Chronic Myeloid Leukemia: many Questions Still Remain to Be Answered. Blood Cancer J. 11, 10–11. 10.1038/s41408-021-00475-7 33927187 PMC8085192

[B59] Eswar N. Webb B. Marti‐Renom M. A. Madhusudhan M. S. Eramian D. Shen M. y. (2006). Comparative Protein Structure Modeling Using Modeller. Curr. Protoc. Bioinformatics 15, 1–30. 10.1002/0471250953.bi0506s15 PMC4186674 18428767

[B61] Everett M. J. Davies D. T. (2021). *Pseudomonas aeruginosa* Elastase (LasB) as a Therapeutic Target. Drug Discov. Today 26, 2108–2123. 10.1016/j.drudis.2021.02.026 33676022

[B62] Evers B. Jastrzebski K. Heijmans J. P. M. Grernrum W. Beijersbergen R. L. Bernards R. (2016). CRISPR Knockout Screening Outperforms shRNA and CRISPRi in Identifying Essential Genes. Nat. Biotechnol. 34, 631–633. 10.1038/nbt.3536 27111720

[B63] Flume P. A. O'Sullivan B. P. Robinson K. A. Goss C. H. Mogayzel P. J. Willey-Courand D. B. (2007). Cystic Fibrosis Pulmonary Guidelines. Am. J. Respir. Crit. Care Med. 176, 957–969. 10.1164/rccm.200705-664OC 17761616

[B64] Foulkes D. M. McLean K. Zheng Y. Sarsby J. Haneef A. S. Fernig D. G. (2021). A Pipeline to Evaluate Inhibitors of the *Pseudomonas aeruginosa* Exotoxin U. Biochem. J. 478, 647–668. 10.1042/BCJ20200780 33459338 PMC7886320

[B66] Fullagar J. L. Garner A. L. Struss A. K. Day J. A. Martin D. P. Yu J. (2013). Antagonism of a Zinc Metalloprotease Using a Unique Metal-Chelating Scaffold: Tropolones as Inhibitors of *P. aeruginosa* Elastase. Chem. Commun. 49, 3197–3199. 10.1039/c3cc41191e PMC3618488 23482955

[B67] Galdino A. C. M. de Oliveira M. P. Ramalho T. C. de Castro A. A. Branquinha M. H. Santos A. L. S. (2019). Anti-Virulence Strategy against the Multidrug-Resistant Bacterial Pathogen *Pseudomonas aeruginosa*: Pseudolysin (Elastase B) as a Potential Druggable Target. Cpps 20, 471–487. 10.2174/1389203720666190207100415 30727891

[B68] Gallagher L. A. McKnight S. L. Kuznetsova M. S. Pesci E. C. Manoil C. (2002). Functions Required for Extracellular Quinolone Signaling by *Pseudomonas aeruginosa* . J. Bacteriol. 184, 6472–6480. 10.1128/JB.184.23.6472-6480.2002 12426334 PMC135424

[B69] Gao X. Mu Z. Qin B. Sun Y. Cui S. (2017). Structure-based Prototype Peptides Targeting the *Pseudomonas aeruginosa* Type VI Secretion System Effector as a Novel Antibacterial Strategy. Front. Cel. Infect. Microbiol. 7. 10.3389/fcimb.2017.00411 PMC5611513 28979890

[B70] Garibyan L. Avashia N. (2013). Polymerase Chain Reaction. J. Invest. Dermatol. 133, 1–4. 10.1038/jid.2013.1 PMC4102308 23399825

[B71] Garner A. L. Struss A. K. Fullagar J. L. Agrawal A. Moreno A. Y. Cohen S. M. (2012). 3-Hydroxy-1-alkyl-2-methylpyridine-4(1H)-thiones: Inhibition of the *Pseudomonas aeruginosa* Virulence Factor LasB. ACS Med. Chem. Lett. 3, 668–672. 10.1021/ml300128f 23181168 PMC3501683

[B72] Gillner D. M. Becker D. P. Holz R. C. (2013). Lysine Biosynthesis in Bacteria: A Metallodesuccinylase as a Potential Antimicrobial Target. J. Biol. Inorg. Chem. 18, 155–163. 10.1007/s00775-012-0965-1 23223968 PMC3862034

[B73] Gimeno A. Ojeda-Montes M. Tomás-Hernández S. Cereto-Massagué A. Beltrán-Debón R. Mulero M. (2019). The Light and Dark Sides of Virtual Screening: What Is There to Know? Ijms 20, 1375. 10.3390/ijms20061375 PMC6470506 30893780

[B74] Goodman C. McFadden G. (2008). Fatty Acid Synthesis in Protozoan Parasites: Unusual Pathways and Novel Drug Targets. Cpd 14, 901–916. 10.2174/138161208784041088 18473839

[B75] Ha U.-H. Wang Y. Jin S. (2003). DsbA of *Pseudomonas aeruginosa* Is Essential for Multiple Virulence Factors. Infect. Immun. 71, 1590–1595. 10.1128/IAI.71.3.1590-1595.2003 12595484 PMC148828

[B76] Hajduk P. J. Olejniczak E. T. Fesik S. W. (1997). One-dimensional Relaxation- and Diffusion-Edited NMR Methods for Screening Compounds that Bind to Macromolecules. J. Am. Chem. Soc. 119, 12257–12261. 10.1021/ja9715962

[B77] Halgren T. A. (2009). Identifying and Characterizing Binding Sites and Assessing Druggability. J. Chem. Inf. Model. 49, 377–389. 10.1021/ci800324m 19434839

[B78] Hauser A. R. (2009). The Type III Secretion System of *Pseudomonas aeruginosa*: Infection by Injection. Nat. Rev. Microbiol. 7, 654–665. 10.1038/nrmicro2199 19680249 PMC2766515

[B79] Heath R. J. Rock C. O. (2006). Fatty Acid Biosynthesis as a Target for Novel Antibacterials. Curr. Opin. Investig. Drugs 5, 146–153. PMC1618763 15043388

[B80] Heidrich J. Sperl L. E. Boeckler F. M. (2019). Embracing the Diversity of Halogen Bonding Motifs in Fragment-Based Drug Discovery-Construction of a Diversity-Optimized Halogen-Enriched Fragment Library. Front. Chem. 7. 10.3389/fchem.2019.00009 PMC6387937 30834240

[B81] Held D. Kilz P. (2021). Size-exclusion Chromatography as a Useful Tool for the Assessment of Polymer Quality and Determination of Macromolecular Properties. Chem. Teach. Int. 3, 77–103. 10.1515/cti-2020-0024

[B82] Heras B. Shouldice S. R. Totsika M. Scanlon M. J. Schembri M. A. Martin J. L. (2009). DSB Proteins and Bacterial Pathogenicity. Nat. Rev. Microbiol. 7, 215–225. 10.1038/nrmicro2087 19198617

[B83] Hoffer L. Voitovich Y. V. Raux B. Carrasco K. Muller C. Fedorov A. Y. (2018). Integrated Strategy for Lead Optimization Based on Fragment Growing: The Diversity-Oriented-Target-Focused-Synthesis Approach. J. Med. Chem. 61, 5719–5732. 10.1021/acs.jmedchem.8b00653 29883107

[B84] Horna G. Ruiz J. (2021a). Type 3 Secretion System as an Anti-pseudomonal Target. Microb. Pathogenesis 155, 104907. 10.1016/j.micpath.2021.104907 33930424

[B85] Horna G. Ruiz J. (2021b). Type 3 Secretion System of *Pseudomonas aeruginosa* . Microbiol. Res. 246, 126719. 10.1016/j.micres.2021.126719 33582609

[B86] Hoseini S. Sauer M. G. (2015). Molecular Cloning Using Polymerase Chain Reaction, an Educational Guide for Cellular Engineering. J. Biol. Eng. 9, 2. 10.1186/1754-1611-9-2 25745516 PMC4350901

[B87] Huang B. Schroeder M. (2006). LIGSITEcsc: Predicting Ligand Binding Sites Using the Connolly Surface and Degree of Conservation. BMC Struct. Biol. 6, 11–19. 10.1186/1472-6807-6-19 16995956 PMC1601958

[B88] Hubbard R. Davis B. Chen I. Drysdale M. (2007). The SeeDs Approach: Integrating Fragments into Drug Discovery. Ctmc 7, 1568–1581. 10.2174/156802607782341109 17979768

[B89] Hubbard R. E. Murray J. B. (2011). Experiences in Fragment-Based lead Discovery. 1st ed. Elsevier, 509–531. 10.1016/B978-0-12-381274-2.00020-0 21371604

[B90] Hueck C. J. (1998). Type III Protein Secretion Systems in Bacterial Pathogens of Animals and Plants. Microbiol. Mol. Biol. Rev. 62, 379–433. 10.1128/mmbr.62.2.379-433.1998 9618447 PMC98920

[B91] Hutton C. A. Perugini M. A. Gerrard J. A. (2007). Inhibition of Lysine Biosynthesis: An Evolving Antibiotic Strategy. Mol. Biosyst. 3, 458–465. 10.1039/b705624a 17579770

[B92] Ibrahim D. Jabbour J.-F. Kanj S. S. (2020). Current Choices of Antibiotic Treatment for *Pseudomonas aeruginosa* Infections. Curr. Opin. Infect. Dis. 33, 464–473. 10.1097/QCO.0000000000000677 33148986

[B93] Irwin J. J. Shoichet B. K. (2005). ZINC − A Free Database of Commercially Available Compounds for Virtual Screening. J. Chem. Inf. Model. 45, 177–182. 10.1021/ci049714+ 15667143 PMC1360656

[B94] Jackman J. E. Fierke C. A. Tumey L. N. Pirrung M. Uchiyama T. Tahir S. H. (2000). Antibacterial Agents that Target Lipid A Biosynthesis in Gram-Negative Bacteria. J. Biol. Chem. 275, 11002–11009. 10.1074/jbc.275.15.11002 10753902

[B95] Jander G. Rahme L. G. Ausubel F. M. (2000). Positive Correlation between Virulence of *Pseudomonas aeruginosa* Mutants in Mice and Insects. J. Bacteriol. 182, 3843–3845. 10.1128/JB.182.13.3843-3845.2000 10851003 PMC94559

[B96] Jenkins R. J. Dotson G. D. (2012). Dual Targeting Antibacterial Peptide Inhibitor of Early Lipid a Biosynthesis. ACS Chem. Biol. 7, 1170–1177. 10.1021/cb300094a 22530734 PMC3401278

[B97] Jenkins R. J. Heslip K. A. Meagher J. L. Stuckey J. A. Dotson G. D. (2014). Structural Basis for the Recognition of Peptide RJPXD33 by Acyltransferases in Lipid a Biosynthesis. J. Biol. Chem. 289, 15527–15535. 10.1074/jbc.M114.564278 24742680 PMC4140908

[B98] Jerabek-Willemsen M. André T. Wanner R. Roth H. M. Duhr S. Baaske P. (2014). MicroScale Thermophoresis: Interaction Analysis and beyond. J. Mol. Struct. 1077, 101–113. 10.1016/j.molstruc.2014.03.009

[B99] Joo S. H. (2015). Lipid A as a Drug Target and Therapeutic Molecule. Biomolecules Ther. 23, 510–516. 10.4062/biomolther.2015.117 PMC4624066 26535075

[B100] Jumper J. Evans R. Pritzel A. Green T. Figurnov M. Ronneberger O. (2021). Highly Accurate Protein Structure Prediction with AlphaFold. Nature 596, 583–589. 10.1038/s41586-021-03819-2 34265844 PMC8371605

[B101] Kalinin D. V. Holl R. (2017). LpxC Inhibitors: a Patent Review (2010-2016). Expert Opin. Ther. Patents 27, 1227–1250. 10.1080/13543776.2017.1360282 28742403

[B102] Kany A. M. Sikandar A. Haupenthal J. Yahiaoui S. Maurer C. K. Proschak E. (2018a). Binding Mode Characterization and Early *In Vivo* Evaluation of Fragment-like Thiols as Inhibitors of the Virulence Factor LasB from *Pseudomonas aeruginosa* . ACS Infect. Dis. 4, 988–997. 10.1021/acsinfecdis.8b00010 29485268

[B103] Kany A. M. Sikandar A. Yahiaoui S. Haupenthal J. Walter I. Empting M. (2018b). Tackling *Pseudomonas aeruginosa* Virulence by a Hydroxamic Acid-Based LasB Inhibitor. ACS Chem. Biol. 13, 2449–2455. 10.1021/acschembio.8b00257 30088919

[B104] Kaya C. Walter I. Yahiaoui S. Sikandar A. Alhayek A. Konstantinović J. (2021). Substrate‐Inspired Fragment Merging and Growing Affords Efficacious LasB Inhibitors. Angew. Chem. Int. Ed. 61, 1–6. 10.1002/anie.202112295 PMC9299988 34762767

[B105] Khattri R. B. Morris D. L. Bilinovich S. M. Manandhar E. Napper K. R. Sweet J. W. (2020). Identifying Ortholog Selective Fragment Molecules for Bacterial Glutaredoxins by NMR and Affinity Enhancement by Modification with an Acrylamide Warhead. Molecules 25, https://www.mdpi.com/1420-3049/25/1/147 . 10.3390/molecules25010147 PMC6983068 31905878

[B106] King J. D. Kocíncová D. Westman E. L. Lam J. S. (2009). Review: Lipopolysaccharide Biosynthesis inPseudomonas Aeruginosa. Innate Immun. 15, 261–312. 10.1177/1753425909106436 19710102

[B107] Koch G. (2017). Composing Compound Libraries for Hit Discovery – Rationality-Driven Preselection or Random Choice by Structural Diversity? Chimia (Aarau) 71, 643. 10.2307/j.ctvnwc0d0.18 25531968

[B108] Konstantinović J. Yahiaoui S. Alhayek A. Haupenthal J. Schönauer E. Andreas A. (2020). N-Aryl-3-mercaptosuccinimides as Antivirulence Agents Targeting *Pseudomonas aeruginosa* Elastase and *Clostridium* Collagenases. J. Med. Chem. 63, 8359–8368. 10.1021/acs.jmedchem.0c00584 32470298 PMC7429951

[B109] Kowalska-Krochmal B. Dudek-Wicher R. (2021). The Minimum Inhibitory Concentration of Antibiotics: Methods, Interpretation, Clinical Relevance. Pathogens 10 (2), 165. https://www.mdpi.com/2076-0817/10/2/165 10.3390/pathogens10020165 33557078 PMC7913839

[B110] Krasowski A. Muthas D. Sarkar A. Schmitt S. Brenk R. (2011). DrugPred: A Structure-Based Approach to Predict Protein Druggability Developed Using an Extensive Nonredundant Data Set. J. Chem. Inf. Model. 51, 2829–2842. 10.1021/ci200266d 21995295

[B111] Krause K. M. Haglund C. M. Hebner C. Serio A. W. Lee G. Nieto V. (2019). Potent LpxC Inhibitors with *In Vitro* Activity against Multidrug-Resistant *Pseudomonas aeruginosa* . Antimicrob. Agents Chemother. 63, e00977–19. 10.1128/AAC.00977-19 31451507 PMC6811409

[B112] Kristam R. Gosu R. (2021). “Selection and Identification of Fragment Library,” in Methods for Fragments Screening Using Surface Plasmon Resonance. Editors Zaheer, S. M. Gosu R., 53–57. 10.1007/978-981-16-1536-8_7

[B113] Kroeck K. G. Sacco M. D. Smith E. W. Zhang X. Shoun D. Akhtar A. (2019). Discovery of Dual-Activity Small-Molecule Ligands of *Pseudomonas aeruginosa* LpxA and LpxD Using SPR and X-ray Crystallography. Sci. Rep. 9, 1–12. 10.1038/s41598-019-51844-z 31664082 PMC6820557

[B114] Krojer T. Talon R. Pearce N. Collins P. Douangamath A. Brandao-Neto J. (2017). TheXChemExplorergraphical Workflow Tool for Routine or Large-Scale Protein-Ligand Structure Determination. Acta Cryst. Sect D Struct. Biol. 73, 267–278. 10.1107/S2059798316020234 28291762 PMC5349439

[B115] Kuzmanic A. Bowman G. R. Juarez-Jimenez J. Michel J. Gervasio F. L. (2020). Investigating Cryptic Binding Sites by Molecular Dynamics Simulations. Acc. Chem. Res. 53, 654–661. 10.1021/acs.accounts.9b00613 32134250 PMC7263906

[B116] Lavecchia A. (2015). Machine-learning Approaches in Drug Discovery: Methods and Applications. Drug Discov. TodayToday 20, 318–331. 10.1016/j.drudis.2014.10.012 25448759

[B117] Leach A. R. (2006). RSC Biomolecular Sciences RSC Publishing Presents an Excerpt from : Structure-Based Drug Discovery an Overview. Mol. Biosyst. 2, 429–446.

[B118] Lee J. Wu J. Deng Y. Wang J. Wang C. Wang J. (2013). A Cell-Cell Communication Signal Integrates Quorum Sensing and Stress Response. Nat. Chem. Biol. 9, 339–343. 10.1038/nchembio.1225 23542643

[B119] Lee S. A. Gallagher L. A. Thongdee M. Staudinger B. J. Lippman S. Singh P. K. (2015). General and Condition-specific Essential Functions ofPseudomonas Aeruginosa. Proc. Natl. Acad. Sci. USA 112, 5189–5194. 10.1073/pnas.1422186112 25848053 PMC4413342

[B120] Lee V. T. Pukatzki S. Sato H. Kikawada E. Kazimirova A. A. Huang J. (2007). Pseudolipasin A Is a Specific Inhibitor for Phospholipase A 2 Activity of *Pseudomonas aeruginosa* Cytotoxin ExoU. Infect. Immun. 75, 1089–1098. 10.1128/IAI.01184-06 17178785 PMC1828555

[B121] Liang S. Thomas S. E. Chaplin A. K. Hardwick S. W. Chirgadze D. Y. Blundell T. L. (2022). Structural Insights into Inhibitor Regulation of the DNA Repair Protein DNA-PKcs. Nature 601, 643–648. 10.1038/s41586-021-04274-9 34987222 PMC8791830

[B122] Liberati N. T. Urbach J. M. Miyata S. Lee D. G. Drenkard E. Wu G. (2006). An Ordered, Nonredundant Library of *Pseudomonas aeruginosa* Strain PA14 Transposon Insertion Mutants. Proc. Natl. Acad. Sci. 103, 2833–2838. 10.1073/pnas.0511100103 16477005 PMC1413827

[B123] Linke P. Amaning K. Maschberger M. Vallee F. Steier V. Baaske P. (2016). An Automated Microscale Thermophoresis Screening Approach for Fragment-Based Lead Discovery. J. Biomol. Screen. 21, 414–421. 10.1177/1087057115618347 26637553 PMC4800460

[B124] Long F. Nicholls R. A. Emsley P. Gražulis S. Merkys A. Vaitkus A. (2017). AceDRG: A Stereochemical Description Generator for Ligands. Acta Cryst. Sect D Struct. Biol. 73, 112–122. 10.1107/S2059798317000067 28177307 PMC5297914

[B125] Lovering F. Bikker J. Humblet C. (2009). Escape from Flatland: Increasing Saturation as an Approach to Improving Clinical success. J. Med. Chem. 52, 6752–6756. 10.1021/jm901241e 19827778

[B126] Lu C. Maurer C. K. Kirsch B. Steinbach A. Hartmann R. W. (2014). Overcoming the Unexpected Functional Inversion of a PqsR Antagonist inPseudomonas Aeruginosa: An *In Vivo* Potent Antivirulence Agent TargetingpqsQuorum Sensing. Angew. Chem. Int. Ed. 53, 1109–1112. 10.1002/anie.201307547 24338917

[B127] Manos J. (2021). Current and Emerging Therapies to Combat Cystic Fibrosis Lung Infections. Microorganisms 9, 1874. 10.3390/microorganisms9091874 34576767 PMC8466233

[B128] Maple H. J. Garlish R. A. Rigau-Roca L. Porter J. Whitcombe I. Prosser C. E. (2012). Automated Protein-Ligand Interaction Screening by Mass Spectrometry. J. Med. Chem. 55, 837–851. 10.1021/jm201347k 22148839

[B129] Mashalidis E. H. Śledź P. Lang S. Abell C. (2013). A Three-Stage Biophysical Screening cascade for Fragment-Based Drug Discovery. Nat. Protoc. 8, 2309–2324. 10.1038/nprot.2013.130 24157549

[B130] Mayer M. Meyer B. (1999). Characterization of Ligand Binding by Saturation Transfer Difference NMR Spectroscopy. Angew. Chem. Int. Ed. 38, 1784–1788. 10.1002/(sici)1521-3773(19990614)38:12<1784::aid-anie1784>3.0.co;2-q 29711196

[B131] Melville J. Burke E. Hirst J. (2009). Machine Learning in Virtual Screening. Cchts 12, 332–343. 10.2174/138620709788167980 19442063

[B132] Miyake Y. Itoh Y. Hatanaka A. Suzuma Y. Suzuki M. Kodama H. (2019). Identification of Novel Lysine Demethylase 5-selective Inhibitors by Inhibitor-Based Fragment Merging Strategy. Bioorg. Med. Chem. 27, 1119–1129. 10.1016/j.bmc.2019.02.006 30745098

[B133] Mohanty B. Rimmer K. McMahon R. M. Headey S. J. Vazirani M. Shouldice S. R. (2017). Fragment Library Screening Identifies Hits that Bind to the Non-catalytic Surface of *Pseudomonas aeruginosa* DsbA1. PLoS One 12, e0173436–20. 10.1371/journal.pone.0173436 28346540 PMC5367682

[B134] Molecular Operating Environment (2016). Integrated Computer-Aided Molecular Design Platform. Available at: https://www.chemcomp.com/Products.htm .

[B135] Molina S. A. Hunt W. R. (2017). Cystic Fibrosis: An Overview of the Past, Present, and the Future. Elsevier Inc., 219–249. 10.1016/B978-0-12-803809-3.00012-9

[B136] Morgens D. W. Deans R. M. Li A. Bassik M. C. (2016). Systematic Comparison of CRISPR/Cas9 and RNAi Screens for Essential Genes. Nat. Biotechnol. 34, 634–636. 10.1038/nbt.3567 27159373 PMC4900911

[B137] Moynie L. Schnell R. McMahon S. A. Sandalova T. Boulkerou W. A. Schmidberger J. W. (2013). The AEROPATH Project targetingPseudomonas Aeruginosa: Crystallographic Studies for Assessment of Potential Targets in Early-Stage Drug Discovery. Acta Cryst. Sect F 69, 25–34. 10.1107/S1744309112044739 PMC3539698 23295481

[B138] Mueller A. M. Breitsprecher D. Duhr S. Baaske P. Schubert T. Längst G. (2017). MicroScale Thermophoresis: A Rapid and Precise Method to Quantify Protein-Nucleic Acid Interactions in Solution. Methods Mol. Biol. 1654, 151–164. 10.1007/978-1-4939-7231-9_10 28986788

[B139] Mullis K. B. (1990). The Unusual Origin of the Polymerase Chain Reaction. Sci. Am. 262, 56–65. 10.1038/scientificamerican0490-56 2315679

[B140] Murray C. W. Rees D. C. (2009). The Rise of Fragment-Based Drug Discovery. Nat. Chem 1, 187–192. 10.1038/nchem.217 21378847

[B141] Murshudov G. N. Skubák P. Lebedev A. A. Pannu N. S. Steiner R. A. Nicholls R. A. (2011). *REFMAC5* for the Refinement of Macromolecular crystal Structures. Acta Crystallogr. D Biol. Cryst. 67, 355–367. 10.1107/S0907444911001314 21460454 PMC3069751

[B142] Müsken M. Di Fiore S. Dötsch A. Fischer R. Häussler S. (2010). Genetic Determinants of *Pseudomonas aeruginosa* Biofilm Establishment. Microbiology 156, 431–441. 10.1099/mic.0.033290-0 19850623

[B143] Navratilova I. Hopkins A. L. (2010). Fragment Screening by Surface Plasmon Resonance. ACS Med. Chem. Lett. 1, 44–48. 10.1021/ml900002k 24900174 PMC4007845

[B144] Nelson S. K. Kelleher A. Robinson G. Reiling S. Asojo O. A. (2013). Structure of 2-Keto-3-Deoxy-D-Manno-Octulosonate-8-Phosphate Synthase fromPseudomonas Aeruginosa. Acta Cryst. Sect F 69, 1084–1088. 10.1107/S1744309113023993 PMC3792661 24100553

[B145] Neumann T. Junker H.-D. Schmidt K. Sekul R. (2007). SPR-based Fragment Screening: Advantages and Applications. Ctmc 7, 1630–1642. 10.2174/156802607782341073 17979772

[B146] Niesen F. H. Berglund H. Vedadi M. (2007). The Use of Differential Scanning Fluorimetry to Detect Ligand Interactions that Promote Protein Stability. Nat. Protoc. 2, 2212–2221. 10.1038/nprot.2007.321 17853878

[B147] Nikiforov P. O. Surade S. Blaszczyk M. Delorme V. Brodin P. Baulard A. R. (2016). A Fragment Merging Approach towards the Development of Small Molecule Inhibitors of *Mycobacterium tuberculosis* EthR for Use as Ethionamide Boosters. Org. Biomol. Chem. 14, 2318–2326. 10.1039/c5ob02630j 26806381 PMC4759522

[B148] Nishino N. Powers J. C. (1980). *Pseudomonas aeruginosa* Elastase. Development of a New Substrate, Inhibitors, and an Affinity Ligand. J. Biol. Chem. 255, 3482–3486. 10.1016/s0021-9258(19)85724-1 6767718

[B149] Norambuena J. Flores R. Cárdenas J. P. Quatrini R. Chávez R. Levicán G. (2012). Thiol/Disulfide System Plays a Crucial Role in Redox Protection in the Acidophilic Iron-Oxidizing Bacterium *Leptospirillum Ferriphilum* . PLoS One 7, e44576. 10.1371/journal.pone.0044576 22970253 PMC3435265

[B150] O'Loughlin C. T. Miller L. C. Siryaporn A. Drescher K. Semmelhack M. F. Bassler B. L. (2013). A Quorum-sensing Inhibitor Blocks *Pseudomonas aeruginosa* Virulence and Biofilm Formation. Proc. Natl. Acad. Sci. 110, 17981–17986. 10.1073/pnas.1316981110 24143808 PMC3816427

[B151] Pan P. Tonge P. Pan P. (2012). Targeting InhA, the FASII Enoyl-ACP Reductase: SAR Studies on Novel Inhibitor Scaffolds. Ctmc 12, 672–693. 10.2174/156802612799984535 PMC4397217 22283812

[B152] Pandurangan A. P. Blundell T. L. (2020). Prediction of Impacts of Mutations on Protein Structure and Interactions: SDM, a Statistical Approach, and mCSM, Using Machine Learning. Protein Sci. 29, 247–257. 10.1002/pro.3774 31693276 PMC6933854

[B153] Pandurangan A. P. Ochoa-Montaño B. Ascher D. B. Blundell T. L. (2017). SDM: A Server for Predicting Effects of Mutations on Protein Stability. Nucleic Acids Res. 45, W229–W235. 10.1093/nar/gkx439 28525590 PMC5793720

[B154] Pang Z. Raudonis R. Glick B. R. Lin T.-J. Cheng Z. (2019). Antibiotic Resistance in *Pseudomonas aeruginosa*: Mechanisms and Alternative Therapeutic Strategies. Biotechnol. Adv. 37, 177–192. 10.1016/j.biotechadv.2018.11.013 30500353

[B155] Papaioannou E. Utari P. Quax W. (2013). Choosing an Appropriate Infection Model to Study Quorum Sensing Inhibition in *Pseudomonas* Infections. Ijms 14, 19309–19340. 10.3390/ijms140919309 24065108 PMC3794835

[B156] Parsons J. B. Rock C. O. (2011). Is Bacterial Fatty Acid Synthesis a Valid Target for Antibacterial Drug Discovery? Curr. Opin. Microbiol. 14, 544–549. 10.1016/j.mib.2011.07.029 21862391 PMC3193581

[B157] Patel D. Bauman J. D. Arnold E. (2014). Advantages of Crystallographic Fragment Screening: Functional and Mechanistic Insights from a Powerful Platform for Efficient Drug Discovery. Prog. Biophys. Mol. Biol. 116, 92–100. 10.1016/j.pbiomolbio.2014.08.004 25117499 PMC4501029

[B158] Pearce N. M. Krojer T. Bradley A. R. Collins P. Nowak R. P. Talon R. (2017). A Multi-crystal Method for Extracting Obscured Crystallographic States from Conventionally Uninterpretable Electron Density. Nat. Commun. 8, 24–29. 10.1038/ncomms15123 28436492 PMC5413968

[B159] Perera T. P. S. Jovcheva E. Mevellec L. Vialard J. De Lange D. Verhulst T. (2017). Discovery and Pharmacological Characterization of JNJ-42756493 (Erdafitinib), a Functionally Selective Small-Molecule FGFR Family Inhibitor. Mol. Cancer Ther. 16, 1010–1020. 10.1158/1535-7163.MCT-16-0589 28341788

[B160] Perumal D. Lim C. S. Sakharkar M. K. (2007). “In Silico Identification of Putative Drug Targets in *Pseudomonas aeruginosa* through Metabolic Pathway Analysis,” in Pattern Recognition in Bioinformatics. PRIB 2007. Lecture Notes in Computer Science. Editors Rajapakse J. C. Schmidt B. Volkert G. (Berlin, Heidelberg: Springer), 4774, 323–336. 10.1007/978-3-540-75286-8_31

[B161] Perumal D. Sakharkar K. R. Tang T. H. Chow V. T. K. Lim C. S. Samal A. (2010). Cloning and Targeted Disruption of Two Lipopolysaccharide Biosynthesis Genes, kdsA and waaG, of *Pseudomonas aeruginosa* PAO1 by Site-Directed Mutagenesis. J. Mol. Microbiol. Biotechnol. 19, 169–179. 10.1159/000322157 21042030

[B162] Pires D. E. V. Ascher D. B. Blundell T. L. (2014). MCSM: Predicting the Effects of Mutations in Proteins Using Graph-Based Signatures. Bioinformatics 30, 335–342. 10.1093/bioinformatics/btt691 24281696 PMC3904523

[B163] Potron A. Poirel L. Nordmann P. (2015). Emerging Broad-Spectrum Resistance in *Pseudomonas aeruginosa* and Acinetobacter Baumannii : Mechanisms and Epidemiology. Int. J. Antimicrob. Agents 45, 568–585. 10.1016/j.ijantimicag.2015.03.001 25857949

[B164] Radoux C. J. Olsson T. S. G. Pitt W. R. Groom C. R. Blundell T. L. (2016). Identifying Interactions that Determine Fragment Binding at Protein Hotspots. J. Med. Chem. 59, 4314–4325. 10.1021/acs.jmedchem.5b01980 27043011

[B165] Raetz C. R. H. Whitfield C. (2002). Lipopolysaccharide Endotoxins. Annu. Rev. Biochem. 71, 635–700. 10.1146/annurev.biochem.71.110601.135414 12045108 PMC2569852

[B166] Rainard J. M. Pandarakalam G. C. McElroy S. P. (2018). Using Microscale Thermophoresis to Characterize Hits from High-Throughput Screening: A European Lead Factory Perspective. SLAS DISCOVERY: Advancing Sci. Drug Discov. 23, 225–241. 10.1177/2472555217744728 PMC5824829 29460707

[B167] Ranjbar M. Behrouz B. Norouzi F. Mousavi Gargari S. L. (2019). Anti-PcrV IgY Antibodies Protect against *Pseudomonas aeruginosa* Infection in Both Acute Pneumonia and Burn Wound Models. Mol. Immunol. 116, 98–105. 10.1016/j.molimm.2019.10.005 31634816

[B168] Roberts R. J. (2005). How Restriction Enzymes Became the Workhorses of Molecular Biology. Proc. Natl. Acad. Sci. 102, 5905–5908. 10.1073/pnas.0500923102 15840723 PMC1087929

[B169] Rock C. O. Jackowski S. (2002). Forty Years of Bacterial Fatty Acid Synthesis. Biochem. Biophysical Res. Commun. 292, 1155–1166. 10.1006/bbrc.2001.2022 11969206

[B170] Rodriguez E. L. Poddar S. Iftekhar S. Suh K. Woolfork A. G. Ovbude S. (2020). Affinity Chromatography: A Review of Trends and Developments over the Past 50 Years. J. Chromatogr. B 1157, 122332. 10.1016/j.jchromb.2020.122332 PMC7584770 32871378

[B171] Rotella D. P. (1997). Antibacterial Agents that Inhibit Lipid A Biosynthesis. Chemtracts 10, 665–668.

[B172] Saint-Criq V. Villeret B. Bastaert F. Kheir S. Hatton A. Cazes A. (2018). *Pseudomonas aeruginosa* LasB Protease Impairs Innate Immunity in Mice and Humans by Targeting a Lung Epithelial Cystic Fibrosis Transmembrane regulator-IL-6-antimicrobial-repair Pathway. Thorax 73, 49–61. 10.1136/thoraxjnl-2017-210298 28790180 PMC5738602

[B173] Šali A. Blundell T. L. (1993). Comparative Protein Modelling by Satisfaction of Spatial Restraints. J. Mol. Biol. 234, 779–815. 8254673 10.1006/jmbi.1993.1626

[B174] Schnell R. Oehlmann W. Sandalova T. Braun Y. Huck C. Maringer M. (2012). Tetrahydrodipicolinate N-Succinyltransferase and Dihydrodipicolinate Synthase from *Pseudomonas aeruginosa*: Structure Analysis and Gene Deletion. PLoS One 7, e31133. 10.1371/journal.pone.0031133 22359568 PMC3281039

[B175] Schuffenhauer A. Ruedisser S. Marzinzik A. Jahnke W. Selzer P. Jacoby E. (2005). Library Design for Fragment Based Screening. Ctmc 5, 751–762. 10.2174/1568026054637700 16101415

[B176] Scoffin R. Slater M. (2015). The Virtual Elaboration of Fragment Ideas : Growing , Merging and Linking Fragments with Realistic Chemistry Drug Discovery , Development & Delivery. Int. Pharm. Ind. 7, 2–5.

[B177] Scott D. E. Coyne A. G. Hudson S. A. Abell C. (2012). Fragment-based Approaches in Drug Discovery and Chemical Biology. Biochemistry 51, 4990–5003. 10.1021/bi3005126 22697260

[B178] Senior A. W. Evans R. Jumper J. Kirkpatrick J. Sifre L. Green T. (2020). Improved Protein Structure Prediction Using Potentials from Deep Learning. Nature 577, 706–710. 10.1038/s41586-019-1923-7 31942072

[B179] Senisterra G. Chau I. Vedadi M. (2012). Thermal Denaturation Assays in Chemical Biology. ASSAY Drug Development Tech. 10, 128–136. 10.1089/adt.2011.0390 22066913

[B180] Senturk S. Shirole N. H. Nowak D. G. Corbo V. Pal D. Vaughan A. (2017). Rapid and Tunable Method to Temporally Control Gene Editing Based on Conditional Cas9 Stabilization. Nat. Commun. 8, 1–10. 10.1038/ncomms14370 28224990 PMC5322564

[B181] Shao X. Xie Y. Zhang Y. Liu J. Ding Y. Wu M. (2020). Novel Therapeutic Strategies for Treating *Pseudomonas aeruginosa* Infection. Expert Opin. Drug Discov. 15, 1403–1423. 10.1080/17460441.2020.1803274 32880507

[B182] Shen S. Kozikowski A. P. (2016). Why Hydroxamates May Not Be the Best Histone Deacetylase Inhibitors-What Some May Have Forgotten or Would rather Forget? ChemMedChem 11, 15–21. 10.1002/cmdc.201500486 26603496 PMC4765907

[B183] Sheremet A. B. Zigangirova N. A. Zayakin E. S. Luyksaar S. I. Kapotina L. N. Nesterenko L. N. (2018). Small Molecule Inhibitor of Type Three Secretion System Belonging to a Class 2,4-Disubstituted-4h-[1,3,4]-Thiadiazine-5-Ones Improves Survival and Decreases Bacterial Loads in an AirwayPseudomonas aeruginosaInfection in Mice. Biomed. Res. Int. 2018, 1–13. 10.1155/2018/5810767 PMC6151375 30276212

[B184] Shrivastava S. Shrivastava P. Ramasamy J. (2018). World Health Organization Releases Global Priority List of Antibiotic-Resistant Bacteria to Guide Research, Discovery, and Development of New Antibiotics. J. Med. Soc. 32, 76–77. 10.4103/jms.jms_25_17

[B185] Silver L. L. (2007). Multi-targeting by Monotherapeutic Antibacterials. Nat. Rev. Drug Discov. 6, 41–55. 10.1038/nrd2202 17159922

[B186] Singh M. Tam B. Akabayov B. (2018). NMR-fragment Based Virtual Screening: A Brief Overview. Molecules 23, 233. 10.3390/molecules23020233 PMC6017141 29370102

[B187] Skurnik D. Roux D. Aschard H. Cattoir V. Yoder-Himes D. Lory S. (2013). A Comprehensive Analysis of *In Vitro* and *In Vivo* Genetic Fitness of *Pseudomonas aeruginosa* Using High-Throughput Sequencing of Transposon Libraries. Plos Pathog. 9, e1003582. 10.1371/journal.ppat.1003582 24039572 PMC3764216

[B188] Sohn M.-J. Zheng C.-J. Kim W.-G. (2008). Macrolactin S, a New Antibacterial Agent with Fab G-Inhibitory Activity from Bacillus Sp. AT28. J. Antibiot. 61, 687–691. 10.1038/ja.2008.98 19168985

[B189] Souers A. J. Leverson J. D. Boghaert E. R. Ackler S. L. Catron N. D. Chen J. (2013). ABT-199, a Potent and Selective BCL-2 Inhibitor, Achieves Antitumor Activity while Sparing Platelets. Nat. Med. 19, 202–208. 10.1038/nm.3048 23291630

[B190] Srivastava V. K. Yadav R. (2019). Isothermal Titration Calorimetry. Elsevier Inc, 125–137. 10.1016/B978-0-12-816548-5.00009-5

[B191] Storz M. P. Maurer C. K. Zimmer C. Wagner N. Brengel C. De Jong J. C. (2012). Validation of PqsD as an Anti-biofilm Target in *Pseudomonas aeruginosa* by Development of Small-Molecule Inhibitors. J. Am. Chem. Soc. 134, 16143–16146. 10.1021/ja3072397 22992202

[B192] Tap W. D. Wainberg Z. A. Anthony S. P. Ibrahim P. N. Zhang C. Healey J. H. (2015). Structure-Guided Blockade of CSF1R Kinase in Tenosynovial Giant-Cell Tumor. N. Engl. J. Med. 373, 428–437. 10.1056/NEJMoa1411366 26222558

[B193] Tasdemir D. Lack G. Brun R. Rüedi P. Scapozza L. Perozzo R. (2006). Inhibition of *Plasmodium Falciparum* Fatty Acid Biosynthesis: Evaluation of FabG, FabZ, and FabI as Drug Targets for Flavonoids. J. Med. Chem. 49, 3345–3353. 10.1021/jm0600545 16722653

[B194] Thomann A. De Mello Martins A. G. G. Brengel C. Empting M. Hartmann R. W. (2016). Application of Dual Inhibition Concept within Looped Autoregulatory Systems toward Antivirulence Agents against *Pseudomonas aeruginosa* Infections. ACS Chem. Biol. 11, 1279–1286. 10.1021/acschembio.6b00117 26882081

[B195] Turner K. H. Wessel A. K. Palmer G. C. Murray J. L. Whiteley M. (2015). Essential Genome ofPseudomonas Aeruginosain Cystic Fibrosis Sputum. Proc. Natl. Acad. Sci. USA 112, 4110–4115. 10.1073/pnas.1419677112 25775563 PMC4386324

[B196] Uk Cystic Fibrosis Registry 2020 Annual Data Report (2021). UK Cystic Fibrosis Registry 2020 Annual Data Report. Registry.

[B197] Urban A. Leipelt M. Eggert T. Jaeger K.-E. (2001). DsbA and DsbC Affect Extracellular Enzyme Formation in *Pseudomonas aeruginosa* . J. Bacteriol. 183, 587–596. 10.1128/JB.183.2.587-596.2001 11133952 PMC94914

[B198] Van Delden C. Iglewski B. H. (1998). http://www.cdc.gov/ncidod/eid/vol4no4/vandelden.htm. Emerg. Infect. Dis. 4, 551–560. 10.3201/eid0404.980405 9866731 PMC2640238

[B199] Van Opijnen T. Camilli A. (2013). Transposon Insertion Sequencing: A New Tool for Systems-Level Analysis of Microorganisms. Nat. Rev. Microbiol. 11, 435–442. 10.1038/nrmicro3033 23712350 PMC3842022

[B200] Walls D. Walker J. M. (2017). Protein Chromatography. Protein Chromatogr. 1485, 423. 10.1007/978-1-4939-6412-3

[B201] Wang T. Birsoy K. Hughes N. W. Krupczak K. M. Post Y. Wei J. J. (2015). Identification and Characterization of Essential Genes in the Human Genome. Science 350, 1096–1101. 10.1126/science.aac7041 26472758 PMC4662922

[B202] Wang X. Choe Y. Craik C. S. Ellman J. A. (2002). Design and Synthesis of Novel Inhibitors of Gelatinase B. Bioorg. Med. Chem. Lett. 12, 2201–2204. 10.1016/S0960-894X(02)00365-7 12127537

[B203] Wartchow C. A. Podlaski F. Li S. Rowan K. Zhang X. Mark D. (2011). Biosensor-based Small Molecule Fragment Screening with Biolayer Interferometry. J. Comput. Aided. Mol. Des. 25, 669–676. 10.1007/s10822-011-9439-8 21660516

[B204] Wei W. Cherukupalli S. Jing L. Liu X. Zhan P. (2020). Fsp3: A New Parameter for Drug-Likeness. Drug Discov. Today 25, 1839–1845. 10.1016/j.drudis.2020.07.017 32712310

[B205] Who (2017). WHO Priority Pathogens List for R&D of New Antibiotics. Available at: https://www.who.int/news/item/27-02-2017-who-publishes-list-of-bacteria-for-which-new-antibiotics-are-urgently-needed .

[B206] Wickramasinghe S. R. Inglis K. A. Urch J. E. Müller S. van Aalten D. M. F. Fairlamb A. H. (2006). Kinetic, Inhibition and Structural Studies on 3-Oxoacyl-ACP Reductase from *Plasmodium Falciparum*, a Key Enzyme in Fatty Acid Biosynthesis. Biochem. J. 393, 447–457. 10.1042/BJ20050832 16225460 PMC1360695

[B207] Williams A. H. Immormino R. M. Gewirth D. T. Raetz C. R. H. (2006). Structure of UDP-N-Acetylglucosamine Acyltransferase with a Bound Antibacterial Pentadecapeptide. Proc. Natl. Acad. Sci. 103, 10877–10882. 10.1073/pnas.0604465103 16835299 PMC1544142

[B208] Winter G. (2010). Xia2: An Expert System for Macromolecular Crystallography Data Reduction. J. Appl. Cryst. 43, 186–190. 10.1107/S0021889809045701

[B209] Wretlind B. Pavlovskis O. R. (1983). *Pseudomonas aeruginosa* Elastase and its Role in pseudomonas Infections. Rev. Infect. Dis. 5 (Suppl. 5), S998–S1004. 10.1093/clinids/5.supplement_5.s998 6419322

[B210] Yadav S. P. Bergqvist S. Doyle M. L. Neubert T. A. Yamniuk A. P. (2012). MIRG Survey 2011: Snapshot of Rapidly Evolving Label-free Technologies Used for Characterizing Molecular Interactions. J. Biomol. Tech. 23, 94–100. 10.7171/jbt.12-2303-002 22942789 PMC3413936

[B211] Yamada Y. Takashima H. Walmsley D. L. Ushiyama F. Matsuda Y. Kanazawa H. (2020). Fragment-Based Discovery of Novel Non-hydroxamate LpxC Inhibitors with Antibacterial Activity. J. Med. Chem. 63, 14805–14820. 10.1021/acs.jmedchem.0c01215 33210531

[B212] Yamaotsu N. Hirono S. (2018). In Silico fragment-mapping Method: a New Tool for Fragment-Based/structure-Based Drug Discovery. J. Comput. Aided. Mol. Des. 32, 1229–1245. 10.1007/s10822-018-0160-8 30196523

[B213] Yang C.-Y. Wang S. (2010). Computational Analysis of Protein Hotspots. ACS Med. Chem. Lett. 1, 125–129. 10.1021/ml100026a 24900186 PMC4007901

[B214] Yang L. Nilsson M. Gjermansen M. Givskov M. Tolker-Nielsen T. (2009). Pyoverdine and PQS Mediated Subpopulation Interactions Involved in *Pseudomonas aeruginosa* Biofilm Formation. Mol. Microbiol. 74, 1380–1392. 10.1111/j.1365-2958.2009.06934.x 19889094

[B215] You Y. Ramachandra S. G. Jin T. (2020). A CRISPR-Based Method for Testing the Essentiality of a Gene. Sci. Rep. 10, 1–8. 10.1038/s41598-020-71690-8 32901070 PMC7478968

[B216] Zelikman S. Aizler Y. Dudkevitch R. Shoshani S. Korenblit S. Burstein D. (2020). Identification and Characterization of New *Pseudomonas aeruginosa* Type III Secretion System Effectors. 9th ILANIT/FISEB Conf, Eilat, Israel, February 17-20, 2020. Available at: https://program.eventact.com/Agenda/Lecture/206800?code=4266825

[B217] Zhang F. Luo S.-Y. Ye Y.-B. Zhao W.-H. Sun X.-G. Wang Z.-Q. (2008). The Antibacterial Efficacy of an Aceraceous Plant [Shantung maple (Acer Truncatum Bunge)] May Be Related to Inhibition of Bacterial β-oxoacyl-acyl Carrier Protein Reductase (FabG). Biotechnol. Appl. Biochem. 51, 73. 10.1042/ba20070255 18208374

[B218] Zhang Y.-M. Lu Y.-J. Rock C. O. (2004). The Reductase Steps of the Type II Fatty Acid Synthase as Antimicrobial Targets. Lipids 39, 1055–1060. 10.1007/s11745-004-1330-3 15726819

[B219] Zhang Y.-M. Rock C. O. (2004). Evaluation of Epigallocatechin Gallate and Related Plant Polyphenols as Inhibitors of the FabG and FabI Reductases of Bacterial Type II Fatty-Acid Synthase. J. Biol. Chem. 279, 30994–31001. 10.1074/jbc.M403697200 15133034

[B220] Zhu T. Cao S. Su P.-C. Patel R. Shah D. Chokshi H. B. (2013). Hit Identification and Optimization in Virtual Screening: Practical Recommendations Based on a Critical Literature Analysis. J. Med. Chem. 56, 6560–6572. 10.1021/jm301916b 23688234 PMC3772997

